# Single-cell histone chaperones patterns guide intercellular communication of tumor microenvironment that contribute to breast cancer metastases

**DOI:** 10.1186/s12935-023-03166-4

**Published:** 2023-12-06

**Authors:** Jindong Xie, Wei Deng, Xinpei Deng, Jie-Ying Liang, Yuhui Tang, Jun Huang, Hailin Tang, Yutian Zou, Huamao Zhou, Xiaoming Xie

**Affiliations:** 1 State Key Laboratory of Oncology in South China, Guangdong Provincial Clinical Research Center for Cancer, Sun Yat-sen University Cancer Center, 651 East Dongfeng Road, Guangzhou, 510060 China; 2 Guangdong Provincial Key Laboratory of Malignant Tumor Epigenetics and Gene Regulation, Department of Medical Oncology, Sun Yat-sen Memorial Hospital, Sun Yat-sen University, Guangzhou, 510000 China; 3 College of Basic Medicine, Shaoyang University, Shaoyang, China; 4 The Affiliated Nanhua Hospital, Hengyang Medical School, University of South China, Hengyang, China

**Keywords:** Single-cell, Histone chaperones, Breast cancer, Tumor microenvironment, Prognosis

## Abstract

**Background:**

Histone chaperones (HCs) are crucial for governing genome stability and gene expression in multiple cancers. However, the functioning of HCs in the tumor microenvironment (TME) is still not clearly understood.

**Methods:**

Self-tested single-cell RNA-seq data derived from 6 breast cancer (BC) patients with brain and liver metastases were reanalyzed by nonnegative matrix factorization (NMF) algorithm for 36 HCs. TME subclusters were observed with BC and immunotherapy public cohorts to assess their prognosis and immune response. The biological effect of HSPA8, one of the HCs, was verified by transwell assay and wound-healing assays.

**Results:**

Cells including fibroblasts, macrophages, B cells, and T cells, were classified into various subclusters based on marker genes. Additionally, it showed that HCs might be strongly associated with biological and clinical features of BC metastases, along with the pseudotime trajectory of each TME cell type. Besides, the results of bulk-seq analysis revealed that TME cell subclusters mediated by HCs distinguished significant prognostic value for BC patients and were relevant to patients’ immunotherapy responses, especially for B cells and macrophages. In particular, CellChat analysis exhibited that HCs-related TME cell subclusters revealed extensive and diverse interactions with malignant cells. Finally, transwell and wound-healing assays exhibited that HSPA8 deficiency inhibited BC cell migration and invasion.

**Conclusions:**

Collectively, our study first dissected HCs-guided intercellular communication of TME that contribute to BC metastases.

**Supplementary Information:**

The online version contains supplementary material available at 10.1186/s12935-023-03166-4.

## Introduction

Breast cancer (BC) is widely regarded as the predominant form of cancer among females [1]. Globally, the annual occurrence of BC is a staggering 1.7 million cases, demanding urgent attention. The diagnosis, prognosis, and treatment of BC have significantly advanced to date with the introduction of contemporary methods. Early detection leads to the successful treatment of BC, but occasionally the illness reoccurs in secondary locations through a process known as “metastasis”, posing a grave danger to the prognosis of patients. Therefore, gaining a more profound comprehension of the molecular mechanisms underlying BC metastasis could potentially lead to the development of innovative approaches for BC prevention and treatment.

Histone chaperones (HCs) are the cornerstone of histone fate. It protects chromosome templates and regulates the storage, transportation, post-translational modification and nucleosome assembly of histones [2]. HCs affect all processes of chromosomes, playing a crucial role in gene expression, gene replication, gene repair, and gene stability. Importantly, histone dysfunction is associated with many diseases, including tumors [3]. Specifically, the close association between histone chaperone and BC metastasis is well-documented [4]. For instance, in the progression of luminal A subtype BC, the high expression of HJURP, a histone chaperone, indicates a higher likelihood of metastasis [5]. There is evidence to suggest that DAXX, one of the HCs, has a tumor inhibitory effect [6].

Currently, increasing evidences have demonstrated the crucial function of the tumor microenvironment (TME) in the tumor advancement and spread. Furthermore, single-cell RNA sequence (scRNA-seq) uncovered the complex intercellular communication between diverse subtypes of TME cells and tumor cells [7, 8]. In addition to the tumor cells, the TME consists of various cell types such as cancer-associated fibroblasts (CAFs), tumor-associated macrophages (TAMs), T cells, and B cells. Notably, recent research conducted by Yin, et al. has demonstrated that the elimination of Mettl3 in myeloid cells promotes tumor growth and metastasis in vivo [9]. Evidences suggest a strong association between histone acetylation patterns and tumor malignant pathways and TME. For instance, it has been observed that high levels of histone acetylation coincide with increased presence of immunosuppressive cells like regulatory T cells (Tregs) and myeloid-derived suppressor cells [10]. Nevertheless, limited studies have been conducted to explore the interaction between HCs associated subtypes of TME cells and tumor cells.

In this study, we examined the impact of HCs on various TME cells, including malignant cells, endothelial cells, mural cells, CAFs, myeloid cells, B cells, and T cells, based on 40,036 scRNA-seq data derived from 6 samples from BC patients with brain and liver metastases. By nonnegative matrix factorization (NMF) clusters of 36 HCs, it was observed that different patterns of HCs in each BC TME cell type subpopulation manifested extensive and diverse communication with tumor epithelial cells and were associated with different immune characteristics, metabolic pathways, transcription characteristics and prognosis. Based on our current understanding, this study uncovers a novel finding that HCs could potentially facilitate intercellular communication between TME cells and tumor cells, thereby playing a role in the BC progression.

## Materials and methods

### Data collection

**Fig. 1:**
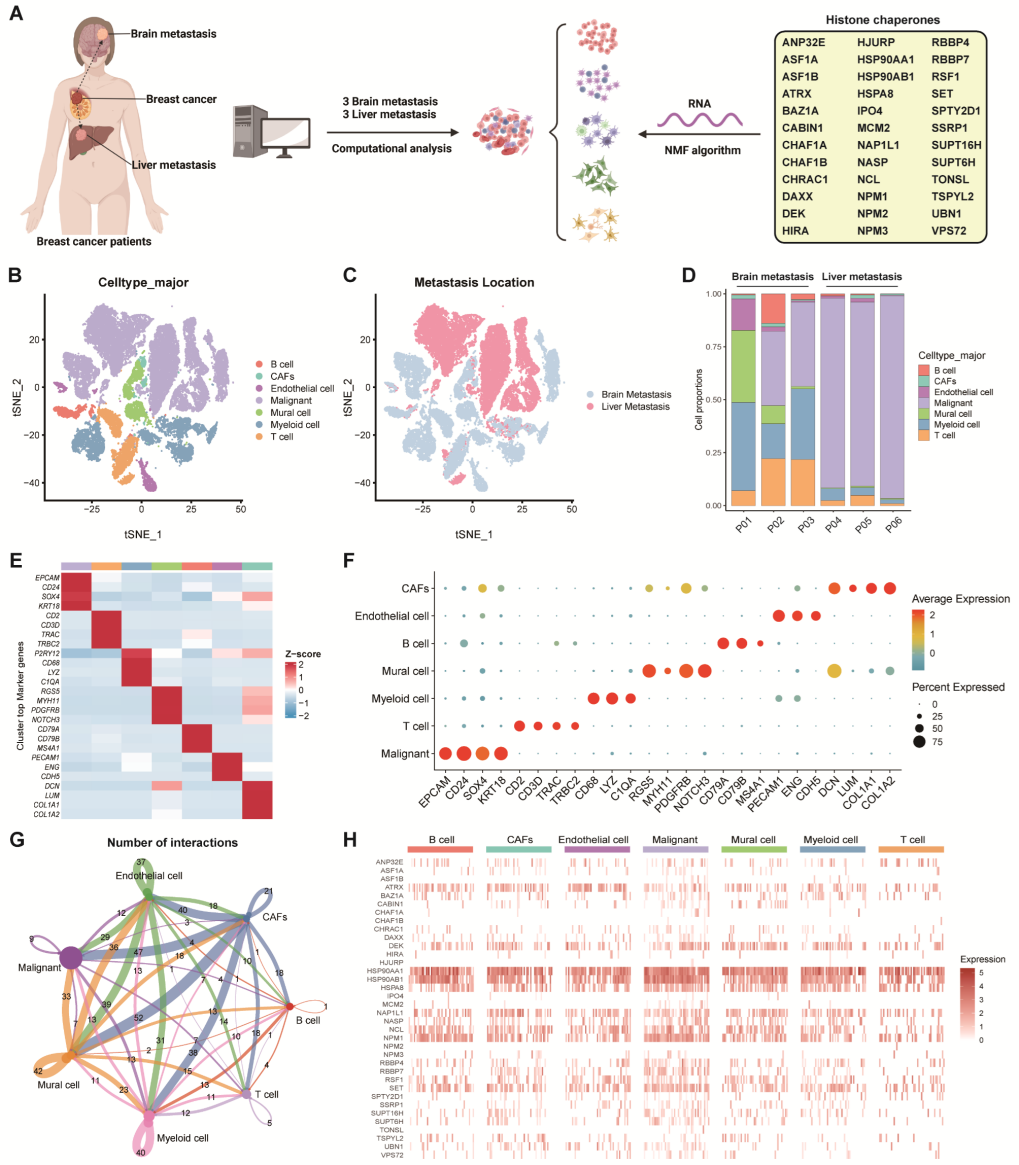
**Overview of HCs in the scRNA-seq data for BC. (A)** The overall design of the present study. **(B)** t-SNE plot of single cells profiled in our previous study colored by major cell type. **(C)** t‐SNE plot of single cells profiled in our previous study colored by metastasis location. **(D)** The composition of each cell type from brain metastasis and liver metastasis patients. **(E)** Heatmap distribution of top marker genes in each cell type. **(F)** Bubble plot of the average and percent expression of top marker genes in each cell type. **(G)** Cell–Cell communications among cell types by Cellchat analysis. **(H)** Heatmap of the expression of HCs in each cell type

The flowchart was shown in Fig. 1A. The research gathered scRNA sequencing data from six BC patients, consisting of three individuals with brain metastasis (BM) and three with liver metastasis (LM). The detailed clinical information of these patients was presented in ***Supplementary Table ******S1***. All samples analyzed in this investigation originated from patients with a confirmed pathological diagnosis of metastatic breast cancer. None of the patients had undergone chemotherapy or radiation treatment for their metastasis before surgery, except for patient P01, who achieved a pathologic complete response following anti-HER2 therapy and chemotherapy. Following initial sample integration, a gene expression and phenotype matrix was generated, encompassing 40,036 scRNA-seq datasets [11]. 36 HCs were collected from a previous study and shown in ***Supplementary Table******S3*** [12]. BC bulk transcriptome and clinical information were collected from the published articles and database (TCGA-BRCA, METABRIC, GSE58812 [13], GSE173661, GSE42568 [14], and GSE103091 [15]). Immune checkpoint blockade immunotherapeutic (ICB) cohorts were collected from the published articles and database (PMID32895571 [16], PMID29301960 [17], GSE91061 [18], GSE35640 [19], GSE145996 [20], GSE126044 [21], GSE115821 [22], and GSE111636). We also collected BC RNA-seq and microarray datasets from bc-GenExMiner database, and multiple scRNA-seq datasets from TISCH database.

### BC visualization of TME cell types and subtypes

The “Seurat” R package was employed to create Seurat objects for both the total and specific cell types within the scRNA-seq gene expression matrix [23]. Subsequently, the 3000 highest-ranking genes, identified as the most variable features, were employed to normalize for each individual cell by utilizing the “FindVariableFeatures” function. Additionally, we executed the “ScaleData” and “RunPCA” functions to determine the principal components count. We employed the t-distributed stochastic neighbor embedding (tSNE) algorithm for dimensionality reduction. Subsequently, leveraging the annotated information from our previous study, we utilized the “Idents” and “DimPlot” functions to annotate and visualize the cells belonging to the predominant cell types or subtypes within the TME.

### Pseudotime trajectory analysis of histone chaperones for TME cells

In order to examine the correlation between cell pseudotime trajectories and HCs, the “Monocle2” R package was utilized [24]. Dimensionality reduction was achieved using the DDR-Tree approach. Afterwards, the “plot_pseudotime_heatmap” function was utilized to create heatmaps that display the changing expression of HCs in the pseudotime trajectories of different TME cell categories in BC.

### NMF of HCs in TME cells

We employed the “NMF” R package and conducted a dimension reduction analysis on 36 HCs across all TME cell types. These procedures were carried out in a manner consistent with previous studies [7, 25].

### Identification of the marker genes of HCs‑mediated cell subtypes in TME cells

The “FindAllMarkers” function was employed to enumerate the markers of each NMF cluster pertaining to every cell type in BC. Additionally, the “AddModuleScore” function computed the signature scores by considering differentially expressed genes (DEGs) across these NMF cell clusters. The distribution of specific signatures of NMF cluster scores in the TME of BC was visualized using the “FeaturePlot” function. The gene sets utilized for comparing the clusters mediated by HCs were collected from MSigDB database and a previous study [26].

### Functional Enrichment Analysis for NMF HCs‑mediated subtypes

The “clusterProfiler” R package was employed to identify potential biological processes using marker genes from various TME cell types within NMF clusters [27]. In addition, we utilized the “scMetabolism” R package to analyze metabolic pathway activity encompassing all cell types in BC [28].

### SCENIC analysis for NMF HCs‑mediated subtypes

The investigation of the gene regulatory network of transcription factors (TFs) in BC utilized the “SCENIC” R package [29]. Two gene-motif rankings, specifically hg19-tss-centered-10 kb and hg19-500 bp-upstream, sourced from the RcisTarget database, were employed to identify the transcription start site and establish the gene regulatory networks in the scRNA-seq data of BC.

### Cell-cell communication analysis for NMF HCs‑mediated subtypes

We employed CellChat with the CellChatDB.human database to assess the primary signaling inputs and outputs within all NMF TME cell clusters [30]. Subsequently, we utilized the “netVisual_circle” function to visually represent the strength or weakness of cell-cell communication networks between the target cell cluster and other cell clusters within the entire set of NMF clusters.

### Survival analyses

Kaplan–Meier (K-M) analyses and Cox regression were performed by “survival” and “survminer” R packages. The cutoff values were calculated by “surv_cutpoint” function. The hazard ratios (HR), the odd ratios (OR) and 95% confidence intervals (CI) were also calculated.

### Cancer immunity cycles and immunoregulation-related pathways

Cancer immunity cycle was derived from previous research [31] and the activities of each step were also estimated [32].

### Cell lines and culture conditions

We used the human epithelial BC cell lines, including MDA-MB-231 and BT549, from the American Type Culture Collection. All cell lines were cultured and maintained according to established protocols, at a temperature of 37 °C and a relative humidity of 99%, without the use of antibiotics. Small interfering RNAs (siRNAs) oligos against HSPA8 were transfected with Lipofectamine 3000 (Invitrogen), and the sequences of the siRNAs used in this study are listed in ***Supplementary Table******S4***.

### Western blot analysis

Cell protein extracts were obtained by utilizing RIPA lysis buffer. Total protein was added to SDS-PAGE and transferred to PVDF membrane from Millipore. Antibody against HSPA8 and β-actin was used. Membrane was incubated with primary antibody at 4 °C overnight, followed by the secondary antibody at room temperature for 1 h. The blots were further visualized with Immobilon Western Chemiluminescent HRP Substrate (Beyotime).

### Transwell assays

A total of 50,000 cells were subjected to digestion and subsequently resuspended. Cells from each experimental group were introduced into the upper chambers, which were devoid of fetal bovine serum (FBS), while the lower cross-pore compartment contained a solution with 20% FBS. Following a 22-hour period, we conducted imaging and quantification of all migrated TNBC cells subsequent to their fixation with methanol and staining with crystal violet (0.1%).

### Cell wound healing assays

The TNBC cells underwent transfection were cultured in 6-well plates at a density of 1 × 10^6^ cells per well for a duration of 24 h. Next, the wounds were generated utilizing a 100 µL pipette tip. The images were captured using a microscope at both 0 and 24 h. We employed image J software to measure the scratch area and assess cell migratory capacity.

### Statistical analysis

The standard statistical tests employed in this study were the Student’s t-test, Wilcoxon rank-sum test, Kruskal–Wallis test, and Chi-square test. These tests were used to assess the differences in continuous target or category variables within the various cell subgroups. Pearson analysis was conducted to examine the correlation between different cell signatures or gene expressions among TME BC cell types. p < 0.05 was considered statistically significant.

## Results

### The landscape of HCs in TME cells in BC

We examined the landscape of HCs using the BC scRNA-seq dataset described previously (Fig. 1A) [11]. We identified 40,036 TME cells in 6 samples from BC patients with brain and liver metastases. These cells were categorized into 7 different types, including malignant cells, endothelial cells, mural cells, CAFs, myeloid cells, B cells, and T cells (Fig. 1B). We also used tSNE to reduce the dimensions and explore the distribution between different metastasis location groups (Fig. 1C). The cell proportions in each patient were also assessed and shown in Fig. 1D and ***Supplementary Table ******S2***. Besides, the annotated cell types were confirmed through the expression of canonical markers and the findings were presented using a heatmap (Fig. 1E), and a bubble plot provided a scaled expression level and proportion of specific markers expressed by each cell type (Fig. 1F). By cell-chat analysis, we also found that these cell types interacted in diverse and distinct manners (Fig. 1G). Moreover, it was clear that HCs were indeed expressed differently in BC metastases according to the scRNA-seq dataset. For example, ASF1A, ASF1B, CHAF1A, CHAF1B, HIRA, HJURP, IPO4, MCM2, NPM2, NPM3, and TONSL exhibited low expression levels in almost all cell types. In contrast, HSP90AA1, HSP90AB1, HSPA8, NAP1L1, NCL, and NPM1 are highly expressed in all cell types (Fig. 1H). Besides, we compared the expression level of HCs between BM and LM and observed that, in BM group, most HCs expression levels are higher than LM in malignant and B cells, whereas are lower in other cell types (***Supplementary Figure ******S1****** and S2***).

### Novel HCs‑mediated CAFs contributed to the TME of BC

**Fig. 2:**
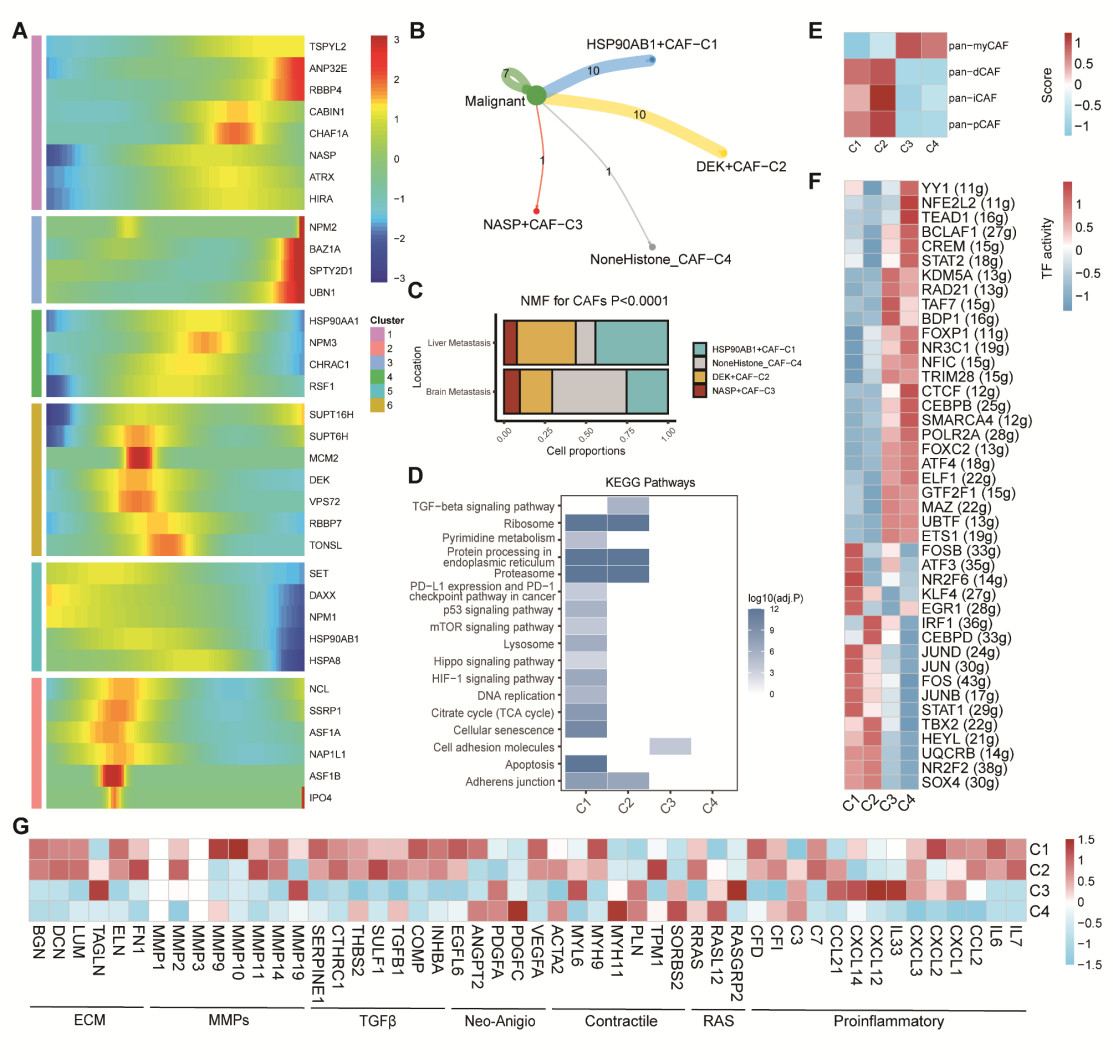
**HCs modified the features of CAFs. (A)** Trajectory analysis revealed the role of HCs in CAFs. **(B)** Cell–Cell communications from HCs-mediated CAFs to malignant cells. **(C)** Bar plot for 4 HCs-mediated CAFs clusters between brain metastasis and liver metastasis patients. **(D)** Heatmap showing the activated KEGG pathways in HCs‑mediated CAFs. **(E)** Different HCs-mediated CAFs clusters were correlated with the previous signatures. **(F)** Heatmap showing the significantly different TFs among HCs-mediated CAFs. **(G)** Heatmap showing the different average expression of common signaling pathway genes in the HCs-mediated CAFs, including collagens, ECM, MMPs, TGFβ, Neo-Angio, Contractile, RAS and Proinflammatory

We first extracted the CAFs subgroup from the scRNA-seq dataset. Based on the pseudotime analysis, we found that the HCs were crucial to CAFs trajectory process (Fig. 2A). By NMF algorithm, we identified 4 HCs‑mediated CAFs subgroups which named as HSP90AB1 + CAF-C1, DEK + CAF-C2, NASP + CAF-C3, and NoneHistone_CAF-C4 (***Supplementary Figure***S3). We then used cell-chat analysis and found that each HCs‑mediated CAFs subgroup had different numbers of ligand-receptor connections, that is, HSP90AB1 + CAF-C1 and DEK + CAF-C2 subgroups had more connections whereas NASP + CAF-C3 and NoneHistone_CAF-C4 possessed less connections (Fig. 2B). Among these subgroups, the HSP90AB1 + CAF-C1 and DEK + CAF-C2 proportions had higher percentages in BC LM samples than that in BM samples while the NASP + CAF-C3 and NoneHistone_CAF-C4 proportions had lower percentages in BC LM samples (Fig. 2C). Besides, the result of the KEGG enrichment analysis showed that the HSP90AB1 + CAF-C1 subgroup was related to numerous classic biological processes such as apoptosis, cellular senescence, TCA cycle, DNA replication, HIF-1 signaling pathway, etc., and the DEK + CAF-C2 subgroup exhibited activities in proteasome, ribosome, and TGF-beta signaling pathway. The NASP + CAF-C3 subgroup was found participated in cell adhesion molecules while the NoneHistone_CAF-C4 did not display a specific biological process (Fig. 2D).

Moreover, we calculated pan-CAF signatures activities among these subgroups, and we found that the HSP90AB1 + CAF-C1 and DEK + CAF-C2 subgroups were obviously correlated with desmoplastic CAF (pan-dCAF), inflammatory CAF (pan-iCAF), and proliferating CAF (pan-pCAF), whereas the NASP + CAF-C3 and NoneHistone_CAF-C4 subgroups were more closely to myofibroblast-like CAF (pan-myCAF) (Fig. 2E). Additionally, analysis of gene regulatory networks among HCs‑mediated CAFs revealed significant differences in TFs. Notably, the HSP90AB1 + CAF-C1 subgroup was characterized by enhanced TF activities of FOS, FOSB, JUN, JUNB, STAT1, etc., and the DEK + CAF-C2 subgroup exhibited upregulated TF activities of IRF1, CEBPD, TBX2, etc. As for the NASP + CAF-C3 and NoneHistone_CAF-C4 subgroups, TF activities like ETS1, ELF1, FOXP1, STAT2 were increasing (Fig. 2F, ***Supplementary Figure***S4). Furthermore, we collected key CAF phenotype markers surface protein genes and compared their expression levels among the HCs-mediated CAFs subgroups. The result indicated that most of them were upregulated in the HSP90AB1 + CAF-C1 and DEK + CAF-C2 subgroups (Fig. 2G).

### HCs‑mediated macrophages/B cells resembled classical characteristics

**Fig. 3:**
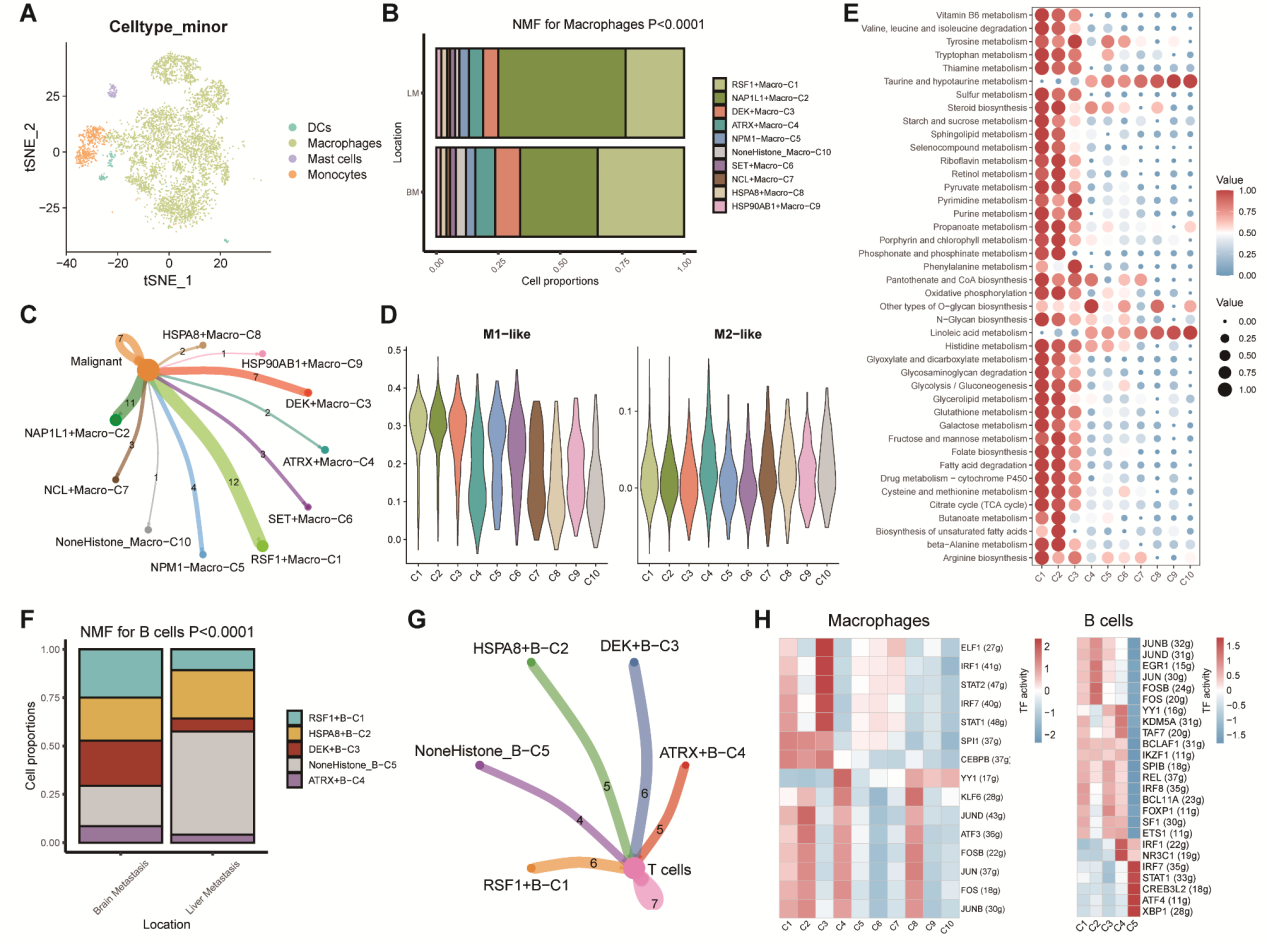
**NMF clusters of HCs for macrophages and B cells. (A)** t-SNE plot of myeloid cells. **(B)** Bar plot for 10 HCs-mediated macrophages clusters between brain metastasis and liver metastasis patients. **(C)** Cell–Cell communications between main HCs-mediated macrophage cells to malignant cells by Cellchat analysis. **(D)** Violin plots of M1 and M2 macrophage-related signatures scores among HCs-mediated macrophages clusters. **(E)** Heatmap showing significantly different metabolic signaling pathways among HCs-mediated macrophages clusters. **(F)** Bar plot for 5 HCs-mediated B cells clusters between brain metastasis and liver metastasis patients. **(G)** Cell–Cell communications between main HCs-mediated B cells to T cells by Cellchat analysis. **(H)** Heatmap showing the significantly different TFs among HCs-mediated macrophages and B cell clusters

Myeloid cells were extracted from the scRNA-seq dataset and split into 4 minor cell types included dendritic cells (DCs), macrophages, mast cells, and monocytes (Fig. 3A). We screened out macrophages and pseudotime analysis also revealed that the HCs were vital to macrophages trajectory process (***Supplementary Figure***S5). We then performed NMF algorithm analysis based on HCs expression. We identified 10 clusters named as RSF1 + Macro-C1, NAP1L1 + Macro-C2, DEK + Macro-C3, ATRX + Macro-C4, NPM1 + Macro-C5, SET + Macro-C6, NCL + Macro-C7, HSPA8 + Macro-C8, HSP90AB1 + Macro-C9, and NoneHistone_ Macro-C10 (***Supplementary Figure***S6). We compared each cluster proportion between liver and BM samples, and we found that the NAP1L1 + Macro-C2 cluster possessed a significantly higher proportion in LM samples while the RSF1 + Macro-C1 cluster was more concentrated in BM samples (Fig. 3B). Similar to CAFs, we also noticed varying connections between HCs‑mediated macrophages and malignant cells, that is, the RSF1 + Macro-C1 cluster, NAP1L1 + Macro-C2 and DEK + Macro-C3 clusters had a large number of links whereas the HSP90AB1 + Macro-C9 and NoneHistone_ Macro-C10 clusters had the less (Fig. 3C). Afterwards, we calculated scores of the macrophage-related signatures in each cluster, and the result showed that the RSF1 + Macro-C1, NAP1L1 + Macro-C2 and DEK + Macro-C3 clusters were significantly associated with M1-like macrophage while the HSP90AB1 + Macro-C9 and NoneHistone_Macro-C10 clusters were strongly related to M2-like macrophage (Fig. 3D). Enrichment analysis also found obvious differences among these clusters (***Supplementary Figure***S7). Besides, we performed SCENIC analysis and found that multiple TFs, such as FOS, FOSB, JUN, JUNB, JUND, etc. were activated in the RSF1 + Macro-C1 and NAP1L1 + Macro-C2 clusters. However, we only observed YY1 activation in the HSP90AB1 + Macro-C9 and NoneHistone_ Macro-C10 clusters (Fig. 3H, ***Supplementary Figure***S8). Previous studies have confirmed that macrophages play an essential role in metabolism. Therefore, we used ssGSEA algorithm to identify the relationship between metabolic pathway activities and each HCs‑mediated macrophage cluster. Interestingly, significant differences were detected among these clusters. The RSF1 + Macro-C1, NAP1L1 + Macro-C2, and DEK + Macro-C3 clusters showed higher metabolic activities in TCA cycle and glycolysis, etc. whereas other clusters fixed on metabolic pathways related to linoleic and taurine/hypotaurine acid metabolism (Fig. 3E).

We also explored B cells heterogeneity based on the result of NMF algorithm analysis. 5 clusters were identified and named as RSF1 + B-C1, HSPA8 + B-C2, DEK + B-C3, ATRX + B-C4, and NoneHistone_ B-C5 (***Supplementary Figure***S9). We found that the proportions of the RSF1 + B-C1 and DEK + Macro-C3 clusters were consistently higher in BM samples, and the NoneHistone_ B-C5 cluster possessed a significantly higher proportion in LM samples (Fig. 3F). Cell-chat analysis showed that HCs‑mediated B cells clusters had similar links to T cells (Fig. 3G). However, the result of enrichment analysis still indicated that the ATRX + B-C4 and NoneHistone_ B-C5 clusters were weakly related to classic biological pathways (***Supplementary Figure***S10). Besides, we still found significant differences among these clusters during SCENIC analysis (Fig. 3H, ***Supplementary Figure***S11).

### HCs‑mediated T cell phenotypes underscored the antitumor immune response in BC

**Fig. 4:**
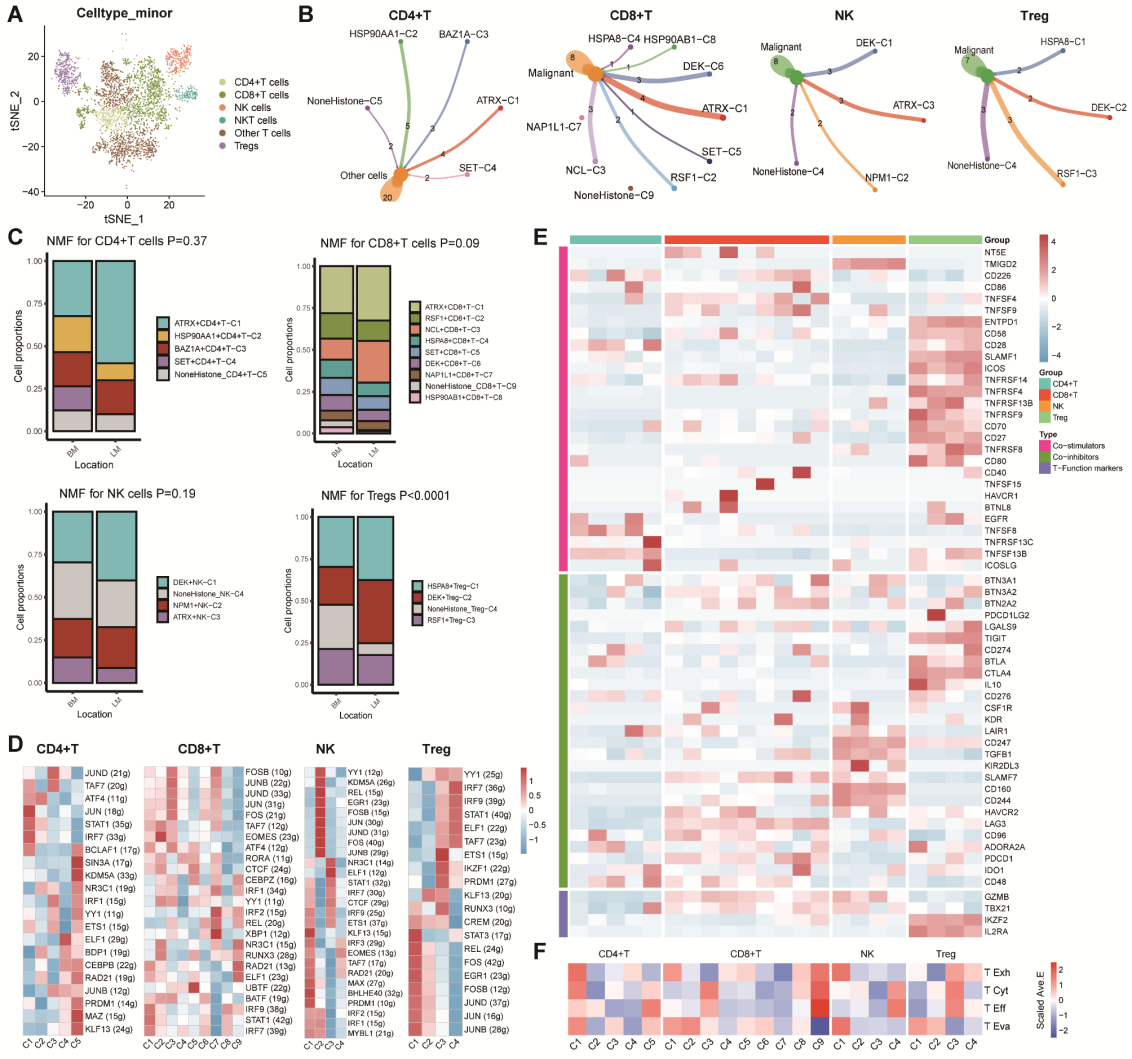
**NMF clusters of HCs for T/NK cells. (A)** t-SNE plot of T/NK cells by six cell types, including CD4 + T cells, CD8 + T cells, Tregs, NK cells, NKT cells, and other T cells. **(B)** Cell–Cell communications from main HCs-mediated T/NK cells to other cells by Cellchat analysis. **(C)** Bar plot of main HCs-mediated T/NK cells clusters between brain metastasis and liver metastasis patients. **(D)** Heatmap showing significantly different TFs among HCs-mediated clusters in CD4 + T cells, CD8 + T cells, NK cells, and Tregs. **(E, F)** Heatmap showing significantly different features among T clusters in CD4 + T cells, CD8 + T cells, NK cells, and Tregs, including immune stimulators, inhibitors and T cell function marker genes, as well as four T function signatures (T exhaustion score, T cytotoxic score, T effector score, and T evasion score)

We renamed 6 main cell types among the detected T cells, including CD4+, CD8+, NK, NKT, Tregs, and other T cells (Fig. 4A). Monocle analyses confirmed that HCs were correlated with T cells trajectory process (***Supplementary Figure***S5). By NMF algorithm analysis, we identified 5 HCs‑mediated CD4 + T clusters (ATRX + CD4 + T-C1, HSP90AA1 + CD4 + T-C2, BAZ1A + CD4 + T-C3, SET + CD4 + T-C4, and NoneHistone_CD4 + T-C5), 9 HCs‑mediated CD8 + T clusters (ATRX + CD8 + T-C1, RSF1 + CD8 + T-C2, NCL + CD8 + T-C3, HSPA8 + CD8 + T-C4, SET + CD8 + T-C5, DEK + CD8 + T-C6, NAP1L1 + CD8 + T-C7, HSP90AB1 + CD8 + T-C8, and NoneHistone_CD8 + T-C9), 4 HCs‑mediated NK clusters (DEK + NK-C1, NPM1 + NK-C2, ATRX + NK-C3, and NoneHistone_NK-C4), and 4 HCs‑mediated Treg clusters (HSPA8 + Treg-C1, DEK + Treg-C2, RSF1 + Treg-C3, and NoneHistone_Treg-C4) (***Supplementary Figure***S12). We performed Cell-chat analyses and found that the NoneHistone_Treg-C4 had more ligand-receptor links compared with other HCs‑mediated Treg clusters, and the NoneHistone clusters in HCs‑mediated CD4 + T, CD8 + T, and NK cells possessed less links (Fig. 4B). We then assessed the proportion of each cluster. Although the proportion of the NoneHistone clusters were consistently higher in BM samples, we only found significant differences in Tregs group (Fig. 4C). Besides, these HCs-mediated T cell phenotypes expressed obvious differences among TFs based on network regulatory analysis (Fig. 4D, ***Supplementary Figure*** S13-16). Moreover, HCs- mediated T clusters were associated with numerous differences in the expression of immune co-inhibitors, co-stimulators, and functional T cell markers (Fig. 4E F).

### HCs‑mediated TME patterns guided Tumor prognosis and immunotherapy

**Fig. 5:**
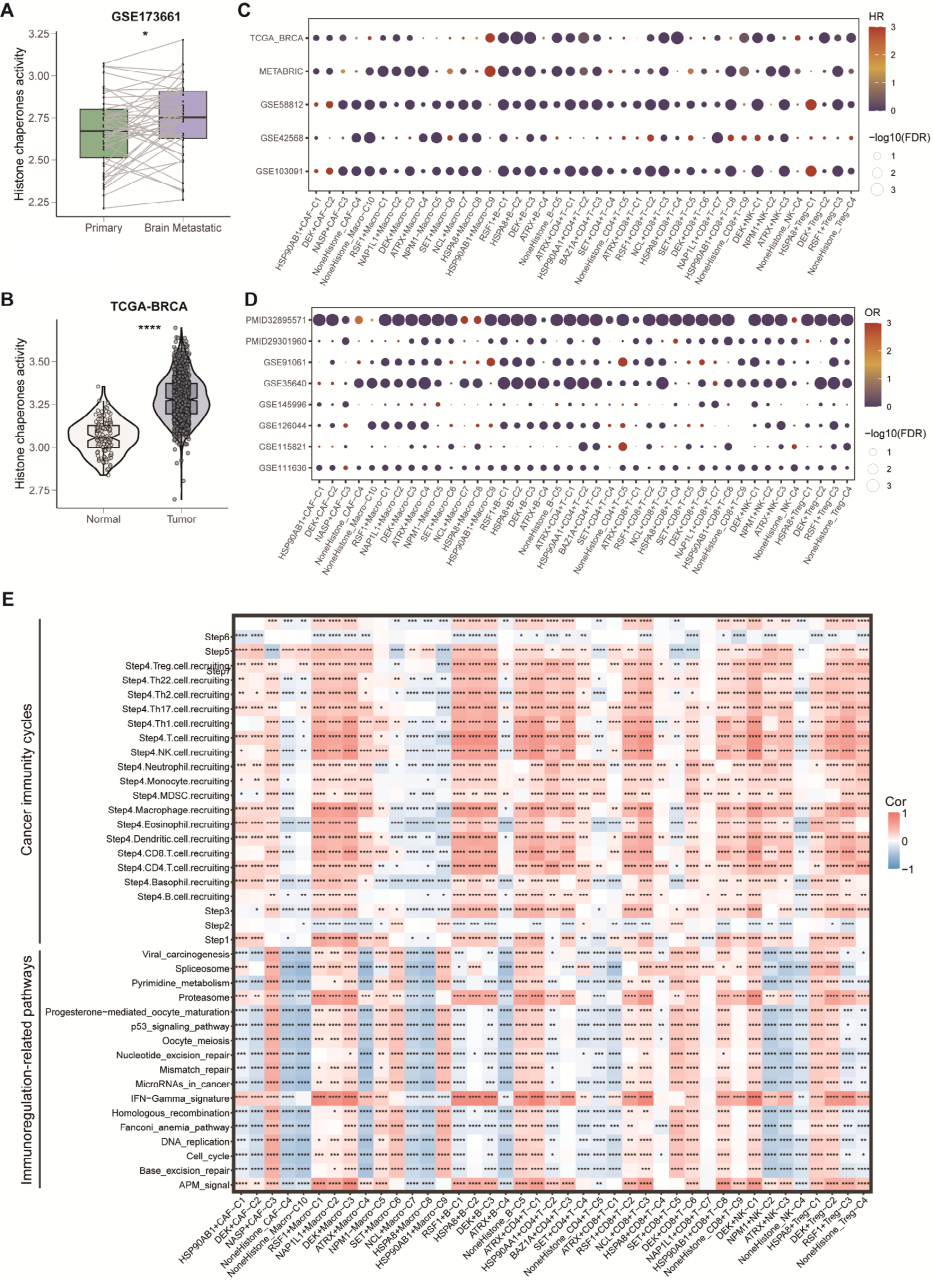
**Overall of the prognosis, immunotherapy response and immunity pathways correlations of HCs-mediated cells types in the public bulk RNA-seq cohorts. (A)** Box plot of HCs activity between primary and paired brain metastatic tissues in GSE173661 cohort (* *p* < 0.05). **(B)** Violin plot of HCs activity between normal and tumor tissues in the TCGA-BRCA cohort (**** *p* < 0.0001). **(C)** Bubble plot of OS analyses (data from 5 BC cohorts). **(D)** Bubble plot of immunotherapy response analyses (data from 8 immunotherapy cohorts with response rate) **(E)** Heatmap showing significant correlations between cancer immunity cycles and immunoregulation-related pathways with all HCs-mediated cluster scores (* p < 0.05, ** *p* < 0.01, *** *p* < 0.001, **** *p* < 0.0001)

Through the utilization of tumor samples and corresponding BM samples sourced from GSE173661, alongside normal and tumor tissues acquired from the TCGA database, our investigation has revealed a notable alteration in the HCs activity score, indicating the significance of HCs in the BC process (Fig. 5A and B). We also calculated the HCs activity scores between BM and LM in each cell type, and we found that the HCs activity scores in BM in malignant and B cells are higher than LM group, whereas are lower in other cell types (***Supplementary Figure***S17). In order to determine the predictive significance of HCs-mediated TME signature, we computed the enrichment score of each HCs-mediated TME cell subtype. Subsequently, the HR for overall survival (OS) was calculated by performing univariate Cox regression analysis for each HCs-related cell subtype in 5 BC cohorts. Notably, we observed significant differences in OS rates among these sub-clusters. For example, HSPA8 + Treg-C1 were identified as unfavorable for BC survival, whereas RSF1 + Macro-C1, NAP1L1 + Macro-C2, DEK + Macro-C3, ATRX + Macro-C4, NoneHistone_Macro-C10, RSF1 + B-C1, HSPA8 + B-C2, DEK + B-C3, ATRX + B-C4, ATRX + CD4 + T-C1, BAZ1A + CD4 + T-C3 and NCL + CD8 + T-C3 were associated with a favorable prognosis in BC (Fig. 5C). Furthermore, in order to forecast the immune response in individuals who received immunotherapy, we employed the logistic regression method to calculate the OR for immune response of each HCs-related cell subtype in 8 ICB cohorts. We observed similar significant phenomena that HCs were relevant to patients’ immunotherapy responses, especially for B cells and macrophages. For example, RSF1 + B-C1, HSPA8 + B-C2, DEK + B-C3, ATRX + B-C4, RSF1 + Macro-C1, NAP1L1 + Macro-C2, DEK + Macro-C3, and ATRX + Macro-C4 were associated with a favorable immunotherapy response in BC (Fig. 5D).

The TME plays a vital role in influencing the immunotherapy effectiveness. Hence, we computed the cancer–immunity cycle scores of BC samples from the TCGA-BRCA. Subsequently, we conducted an analysis to examine the associations between the enrichment score of each HCs-mediated TME cell subtype and the cancer–immunity cycle scores. Notably, the levels of different anti-cancer immune responses, including the release of cancer cell antigens, T cell recruiting, CD8 T cell recruiting, Th1 cell recruiting, NK cell recruiting, and killing of cancer cells, were observed to be significantly elevated in RSF1 + Macro-C1, NAP1L1 + Macro-C2, DEK + Macro-C3, RSF1 + B-C1, HSPA8 + B-C2 and DEK + B-C3, etc. (Fig. 5E). We also computed the immunoregulation-related pathways scores of BC samples from the TCGA-BRCA dataset as well as examined the associations between the enrichment score of each HCs-mediated TME cell subtype with it, and we found similar significant phenomena that immunoregulation-related pathways scores were significantly elevated in RSF1 + Macro-C1, NAP1L1 + Macro-C2, DEK + Macro-C3, whereas were decreased in NCL + Macro-C7, HSPA8 + Macro-C8, and NoneHistone_Macro-C10, indicating that HCs might play an important role in TME (Fig. 5E).

### HSPA8 deficiency inhibits Tumor cell migration and invasion

**Fig. 6:**
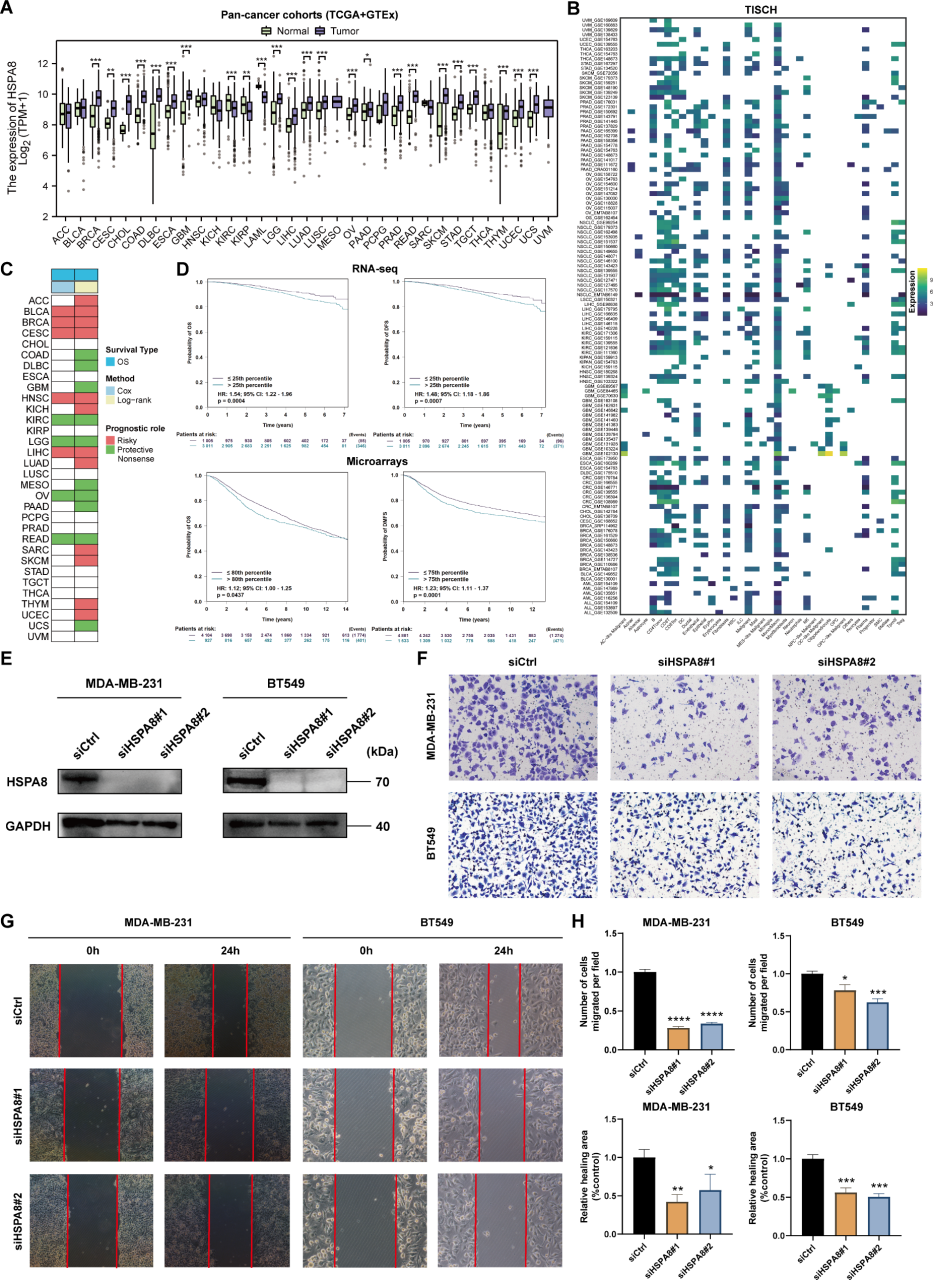
**HSPA8 deficiency inhibits tumor cell migration and invasion. (A)** Boxplots of the HSPA8 expression between tumor and normal tissues in the TCGA pan-cancer cohorts (* p < 0.05, ** p < 0.01, *** p < 0.001). **(B)** Heatmap of the HSPA8 expression among different cell types in TISCH database. **(C)** Heatmap of the prognostic value of HSPA8 in the TCGA pan-cancer cohorts. **(D)** Survival analyses of HSPA8 using K-M analyses in BC RNA-seq and microarray datasets from bc-GenExMiner database. **(E)** Western blot assays showing the efficacy of siRNAs targeting HSPA8 in BC cell lines. **(F)** Transwell migration assays were performed to measure the migration abilities of HSPA8 in BC cell lines. **(G)** Wound healing assays were performed to measure the migration abilities of HSPA8 in BC cell lines. **(H)** Boxplots of the number of cells migrated per field and relative healing area (% control) in BC cell lines (* *p* < 0.05, ** *p* < 0.01, *** *p* < 0.001, **** *p* < 0.0001). ACC, adrenocortical carcinoma; BLCA, bladder urothelial carcinoma; BRCA, breast invasive carcinoma; CESC, cervical squamous cell carcinoma and endocervical adenocarcinoma; CHOL, cholangiocarcinoma; COAD, colon adenocarcinoma; DLBC, lymphoid neoplasm diffuse large B-cell lymphoma; ESCA, esophageal carcinoma; GBM, glioblastoma multiforme; HNSC, head and neck squamous cell carcinoma; KICH, kidney chromophobe; KIRC, kidney renal clear cell carcinoma; KIRP, kidney renal papillary cell carcinoma; LAML, acute myeloid leukemia; LGG, brain lower grade glioma; LIHC, liver hepatocellular carcinoma; LUAD, lung adenocarcinoma; LUSC, lung squamous cell carcinoma; MESO, mesothelioma; OV, ovarian serous cystadenocarcinoma; PAAD, pancreatic adenocarcinoma; PCPG, pheochromocytoma and paraganglioma; PRAD, prostate adenocarcinoma; READ, rectum adenocarcinoma; SARC, sarcoma; SKCM, skin cutaneous melanoma; STAD, stomach adenocarcinoma; TGCT, testicular germ cell tumors; THCA, thyroid carcinoma; THYM, thymoma; UCEC, uterine corpus endometrial carcinoma; UCS, uterine carcinosarcoma; UVM, ocular melanomas. ALL, acute lymphoblastic leukemia; AML, acute myeloid leukemia; KIPAN, pan-kidney cancer; LSCC, laryngeal squamous cell carcinoma; OS, osteosarcoma

In order to investigate the impact of HCs on tumor cells, we specifically chose the HSPA8 to examine its potential tumorigenic effect. We firstly explore its expression difference between tumor and normal tissues from the combination of the TCGA and GTEx databases. We found that HSPA8 was significantly upregulated in most cancer type while significantly downregulated in kidney renal clear cell carcinoma (KIRC), kidney renal papillary cell carcinoma (KIRP), and acute myeloid leukemia (LAML) (Fig. 6A). Then, we assessed the expression level of HSPA8 at the single-cell level, and Fig. 6B showed that HSPA8 widely distributed in various cell type. Next, we performed log-rank and Cox regression analyses to investigate the prognostic role of HSPA8, and we found the its heterogeneity in the prognostic value, that is, HSPA8 was a risk factor in BLCA, BRCA, CESC, HNSC, LIHC, etc. while was a protective factor in KIRC, LGG, OV, READ, etc. (Fig. 6C). Besides, we validated its prognostic value in BC RNA-seq and microarray datasets from bc-GenExMiner database, and the results were consistent with our findings (Fig. 6D). To evaluate the involvement of HSPA8 in the in vitro metastatic behavior of MDA-MB-231 and BT-549 cells, we employed siRNA (HSAP8) transfection to knock down HSPA8 expression. The expression levels of HSPA8 proteins were effectively decreased in MDA-MB-231 and BT-549 cells when compared to untransfected cells (Fig. 6E). As Fig. 6F H shown, knockdown of HSAP8 significantly decreased the migratory capacity of MDA-MB231 and BT-549 cells during transwell and wound healing assays.

## Discussion

To date, numerous studies have elucidated the association between HCs and the etiology of BC [4–6]. Nevertheless, limited research has been conducted on the potential tumorigenic function of HCs at the single-cell levels. In this current investigation, we have undertaken a comprehensive examination of HCs in the primary cell types within TME of BC. Furthermore, we have identified the diverse cell-to-cell interactions between TME cell subtypes associated with HCs and tumor cells, which represents the first instance of such an analysis. This novel and distinct viewpoint has provided us with insights into the impact of HCs on various cellular constituents within TME and its influence on the outcomes of individual BC patients.

The predominant composition of tumor tissue comprises cancer epithelial cells, which play a pivotal role in tumor progression. Moreover, the heterogeneity observed among cancer epithelial cells signifies the varying response to treatment and ultimately determines the prognosis of patients. In addition to cancer epithelial cells, TME cells, including diverse stromal cells and infiltrating immune cells, collectively contribute to tumor growth and facilitate immune evasion in solid tumors [33]. In our study, we observed that various cells within TME, including CAFs, macrophages, T cells, and B cells, exhibited diverse HC patterns and actively communicated with tumor epithelial cells, which was evidenced by scRNA-seq analysis.

CAFs which are considered crucial constituents of stromal cells, have been categorized into pan-dCAFs, pan-myCAFs, pan-iCAFs, and pan-pCAFs [34, 35]. However, limited research has been conducted thus far to investigate the potential involvement of HCs in CAFs. Our study indicates that the NASP + CAF-C3 and NoneHistone_CAF-C4 subgroups exhibit a closer association with pan-myCAF, while the HSP90AB1 + CAF-C1 and DEK + CAF-C2 subgroups are significantly correlated with pan-pCAF, pan-dCAF, and pan-iCAF. Besides, we proposed that histone chaperones may exert an influence on the functionality and phenotype of CAFs. This, in turn, could promote the development of an immunosuppressive TME, ultimately expediting the malignant progression and metastasis of BC. We observe that the HSP90AB1 + CAF-C1 and DEK + CAF-C2 subgroups demonstrate a strong association with increased expression of TGF-β and inflammatory factors such as CXCL1, CXCL3, CCL2, IL-6, IL-7, indicating that these two subgroups might contribute to the formation of an immunosuppressive microenvironment through the secretion of CXCL1, IL6, CCL2 and TGF-β [36–39]. Pathway analysis further reveals the TGF-β signaling pathway in the DEK + CAF-C2 subgroup and the expression of PD-L1 and the PD1 signaling pathway in HSP90AB1 + CAF-C1, which may contribute to an immune-suppressive effect on T cell activation [40]. Consequently, we hypothesize that HC-mediated CAFs may establish an immunosuppressive interaction with tumor cells, thereby facilitating tumor progression and metastasis. Moreover, we observed a strong correlation between the C1 and C2 subgroups and the upregulation of extracellular matrix (ECM) factors and matrix metalloproteinases (MMPs) such as FN1, ELN, MMP9, MMP14, and MMP11. Numerous recent studies consistently indicate that activated fibroblasts exert control over the progression and metastasis of cancer through their active secretory protein, comprising MMP11, MMP14, and FN1 [41–45].

Macrophages, widely distributed throughout the body, play a crucial role in maintaining homeostasis and defending against pathogen intrusion. Macrophages in various tissues undergo polarization in response to environmental changes, resulting in distinct macrophage subtypes, such as M1 and M2 macrophages [10, 46]. Notably, M2 macrophages play a vital role in promoting tumor growth and metastasis and are closely associated with unfavorable prognoses in neoplastic conditions. In contrast, M1 macrophages are commonly regarded as macrophages with tumor-killing properties, primarily functioning in anti-tumor activities and immune promotion [47]. Our findings indicate that the RSF1 + Macro-C1, NAP1L1 + Macro-C2, and DEK + Macro-C3 clusters exhibit higher scores of M1 macrophage-related signatures compared to the remaining Macro-subtypes. Conversely, the HSP90AB1 + Macro-C9 and NoneHistone_Macro-C10 clusters demonstrate a strong association with M2-like macrophages. Furthermore, we have observed variations in the number of connections between HCs-mediated macrophages and malignant cells. Specifically, the RSF1 + Macro-C1, NAP1L1 + Macro-C2, and DEK + Macro-C3 clusters display a larger number of connections, while the HSP90AB1 + Macro-C9 and NoneHistone_Macro-C10 clusters exhibit a lower number of connections. The analysis of pathways also indicated the involvement of TAMs in the tricarboxylic acid (TCA) cycle and glycolysis signaling pathway in the RSF1 + Macro-C1, NAP1L1 + Macro-C2, and DEK + Macro-C3 clusters. M1-like macrophages are commonly linked to an extensively glycolytic metabolism and a robust ability to produce reactive oxygen species, which forms the basis of their cytotoxic activities [48, 49]. We also found that the enrichment score of RSF1 + Macro-C1, NAP1L1 + Macro-C2, and DEK + Macro-C3 clusters have a positive correlation with Cancer immunity cycles score and immunoregulation-related pathways score which indicated a high activity level of all cancer immunity steps [31, 32].

For Treg cells, we observed that the NoneHistone clusters exhibited a greater number of ligand-receptor connections compared to other Treg clusters mediated by HCs, while the NoneHistone clusters in HCs-mediated macrophages, B cells, CD4 + T cells, CD8 + T cells, and NK cells displayed fewer connections. The prevalence of NoneHistone clusters was consistently higher in BM samples, with the exception of B cells. Additionally, the four main T cell subtypes mediated by HCs displayed varying levels of T cell activity and inactivity. For example, ATRX-CD4 + T-C1cluster has a high score of four T function signatures and low expression of T cell inhibitors and T cell function marker genes, DEK-NK-C1 cluster has high level of T cytotoxic score and T effector score, and NoneHistone-NK-C4 cluster has high level of T exhaustion score and T evasion score. These findings collectively suggest the significant involvement of HCs in evading the immune system and the role of macrophages and T cells in promoting tumor growth.

In order to determine the gene regulatory networks specific to each cell type, we analyzed TFs at the scRNA-seq level. Overall, each subtype of CAFs, macrophages, B cells, and various T cell types exhibited distinct characteristics in terms of transcription factors. For CAFs, the subgroups HSP90AB1 + CAF-C1 and DEK + CAF-C2 displayed a distinctive transcription factor gene signature, including KLF4, which facilitated the transcription of CH25H. This resulted in the inhibition of extracellular vesicle uptake by NFs, thereby impeding the conversion of NF to CAFs [50]. Furthermore, the NoneHistone_CAF-C4 subgroups exhibited heightened TEAD1 activity, which promoted the conversion of NF to CAFs [51].

Additionally, we observed distinct transcription factor characteristics in HCs-mediated cell subtypes for B and T cells. For instance, HSPA8 + Treg-C1 and DEK + Treg-C2 exhibited a distinct TF gene signature, such as JUNB, which alerts Tregs of the developing Teff activation and coordinates immune regulation to maintain the immunosuppressive effect of Treg [52]. On the other hand, C3 and C4 exhibited heightened activity of YY1 and STAT1, with the former acting as an inhibitory factor for Treg’s immunosuppressive function, while abnormal expression levels of the latter can result in dysfunction of Treg-mediated immune suppression [53, 54]. In summary, different types of cells facilitated by HCs might have an impact on specific TF regulatory systems, leading to the restructuring and reprogramming of the TME.

In light of the intricate intrinsic patterns exhibited by HCs in TME cells, we conducted a comprehensive analysis to summarize the associations between the scores of these subclusters and their impact on both prognosis and immune response. We observed significant prognostic disparities in BC patients based on the varying dominance of HCs in TME cells by utilizing multiple bulk RNA-seq cohorts. Furthermore, we identified a notable distinction in immune response among patients who underwent immune checkpoint blockade (ICB) therapy, particularly in B cells and macrophages. These findings underscore the crucial role of TME HCs in the context of BC and warrant further investigation.

HSPA8, one of the HCs, was chosen as the subject of an investigation to the processes of tumor metastasis. Pan-cancer analysis revealed the oncogenic nature of HSPA8 in a majority of tumors, including BC. Furthermore, our in vitro experiments yielded evidence indicating that HSPA8 functions as an oncogenic gene, facilitating tumor metastasis and invasion. Consequently, this discovery presents a novel avenue for future cancer prevention and treatment, offering a potential target and therapeutic strategy.

## Conclusions

In this study, we employed the scRNA-seq analysis method to successfully identify distinct HCs-mediated cell subtypes of TME cells. Furthermore, we elucidated the role of HCs-mediated intercellular communication in regulating tumor growth and antitumor immunomodulatory processes, serving as important prognostic markers and indicators of ICB efficacy in BC cohorts.

### Electronic supplementary material

Below is the link to the electronic supplementary material.

Supplementary Material 1

Supplementary Material 2

## Data Availability

All datasets involved in this study can be viewed in the Molecular Signature Database (MSigDB) (https://www.gsea-msigdb.org/gsea/msigdb/), Gene Expression Omnibus (GEO), GenExMiner (http://bcgenex.ico.unicancer.fr/BC-GEM/GEM-requete.php), TISCH (http://tisch.comp-genomics.org/), or data availability part of the corresponding articles.

## Abbreviations

BC: Breast cancer
HC: Histone chaperone
TME: Tumor microenvironment
scRNA-seq: Single-cell RNA sequence
CAFs: Cancer-associated fibroblasts
TAMs: Tumor-associated macrophages
Tregs: Regulatory T cells
NMF: Nonnegative matrix factorization
BM: Brain metastasis
LM: Liver metastasis
tSNE: t-distributed stochastic neighbor embedding
DEGs: Differentially expressed genes
TFs: Transcription factors
K-M: Kaplan–Meier
HR: Hazard ratios
OR: Odd ratios
CI: Confidence intervals
siRNAs: Small interfering RNAs
FBS: Fetal bovine serum
pan-dCAF: Desmoplastic CAF
pan-iCAF: Inflammatory CAF
pan-pCAF: Proliferating CAF
DCs: Dendritic cells. ACC,adrenocortical carcinoma
BLCA: Bladder urothelial carcinoma
BRCA: Breast invasive carcinoma
CESC: Cervical squamous cell carcinoma and endocervical adenocarcinoma
CHOL: Cholangiocarcinoma
COAD: Colon adenocarcinoma
DLBC: Lymphoid neoplasm diffuse large B-cell lymphoma
ESCA: Esophageal carcinoma
GBM: Glioblastoma multiforme
HNSC: Head and neck squamous cell carcinoma
KICH: Kidney chromophobe
KIRC: Kidney renal clear cell carcinoma
KIRP: Kidney renal papillary cell carcinoma
LAML: Acute myeloid leukemia
LGG: Brain lower grade glioma
LIHC: Liver hepatocellular carcinoma
LUAD: Lung adenocarcinoma
LUSC: Lung squamous cell carcinoma
MESO: Mesothelioma
OV: Ovarian serous cystadenocarcinoma
PAAD: Pancreatic adenocarcinoma
PCPG: Pheochromocytoma and paraganglioma
PRAD: Prostate adenocarcinoma
READ: Rectum adenocarcinoma
SARC: Sarcoma
SKCM: Skin cutaneous melanoma
STAD: Stomach adenocarcinoma
TGCT: Testicular germ cell tumors
THCA: Thyroid carcinoma
THYM: Thymoma
UCEC: Uterine corpus endometrial carcinoma
UCS: Uterine carcinosarcoma
UVM: Ocular melanomas. ALL,acute lymphoblastic leukemia
AML: Acute myeloid leukemia
KIPAN: Pan-kidney cancer
LSCC: Laryngeal squamous cell carcinoma
OS: Osteosarcoma
