# Supplementary material for: Single-cell histone chaperones patterns guide intercellular communication of tumor microenvironment that contribute to breast cancer metastases
Source: Cancer Cell Int. 2023 Dec 6;23:311. doi: 10.1186/s12935-023-03166-4 (PMC10702093; doi:10.1186/s12935-023-03166-4)
Supplement: Supplementary file 1 — Supplementary Material 1 [file 12935_2023_3166_MOESM1_ESM.pdf]

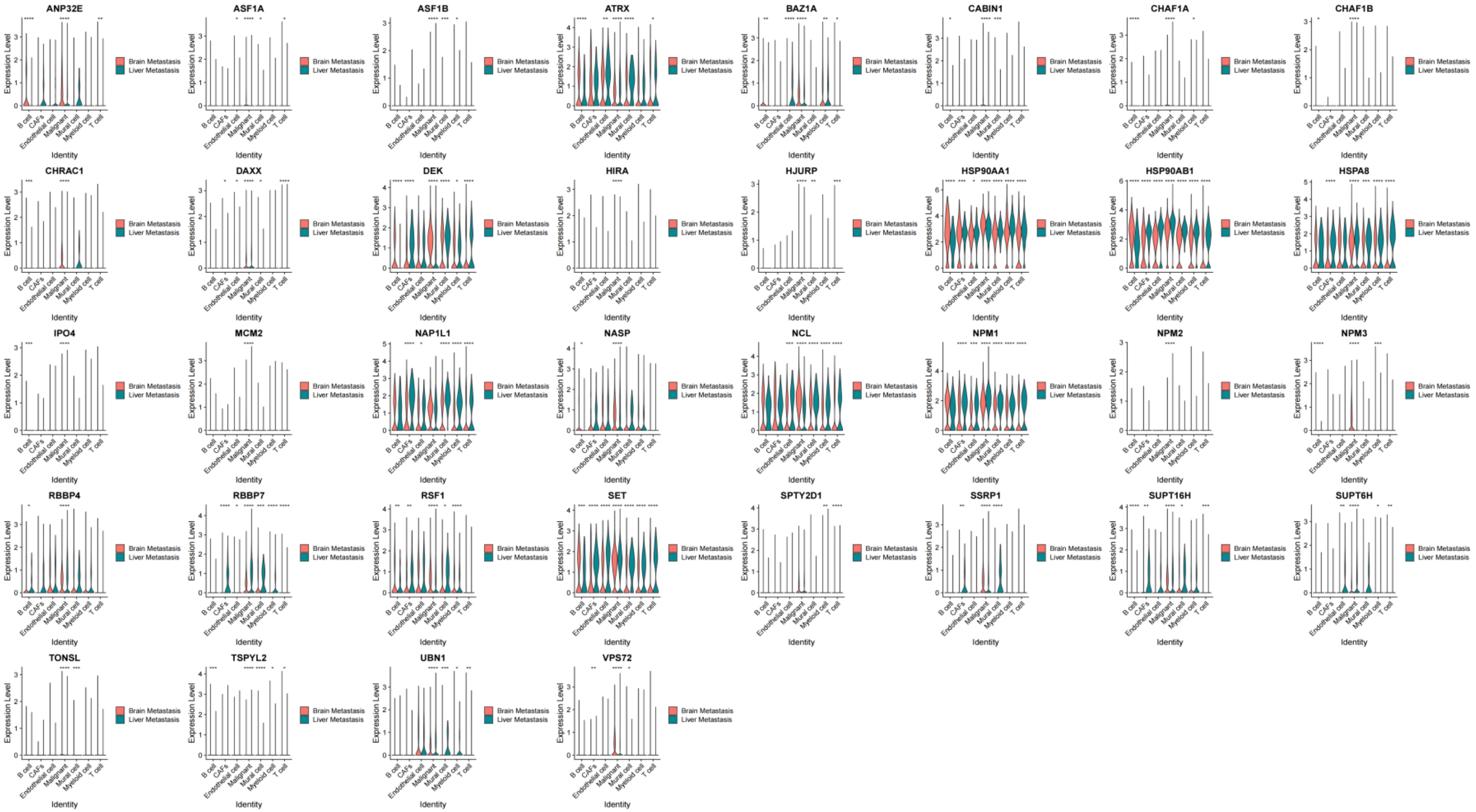

Supplementary Figure S1. Violin plots of HCs expression levels among cell types between brain metastasis and liver metastasis (* p < 0.05, ** p < 0.01, *** p < 0.001, **** p < 0.0001).

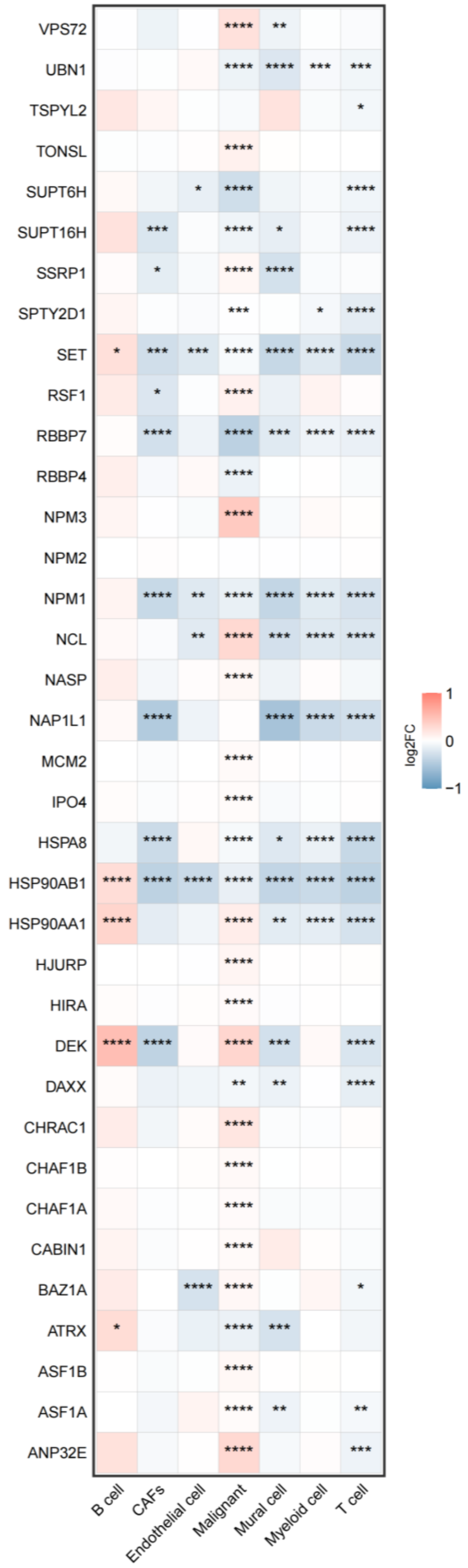

Supplementary Figure S2. Heatmap of HCs expression levels between brain metastasis and liver metastasis in each cell type (FC, Fold change; * p < 0.05, ** p < 0.01, *** p < 0.001, **** p < 0.0001).

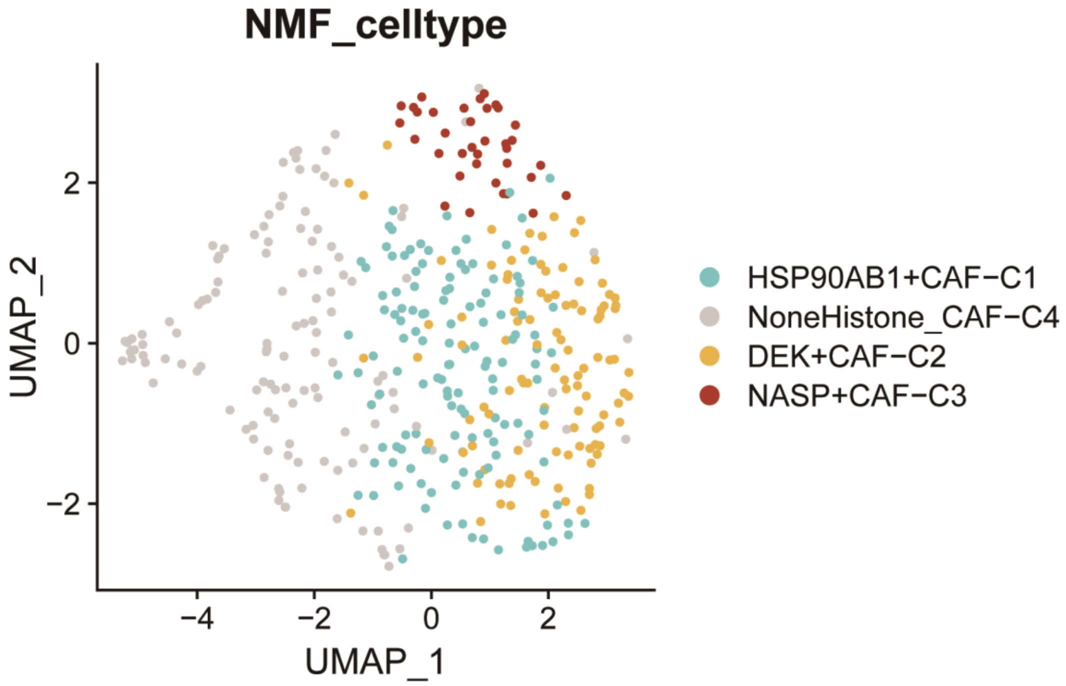

Supplementary Figure S3. UMAP plot for CAFs by 4 cell types, including HSP90AB1+CAF-C1, DEK+CAF-C2, NASP+CAF-C3, and NoneHistone_CAF-C4.

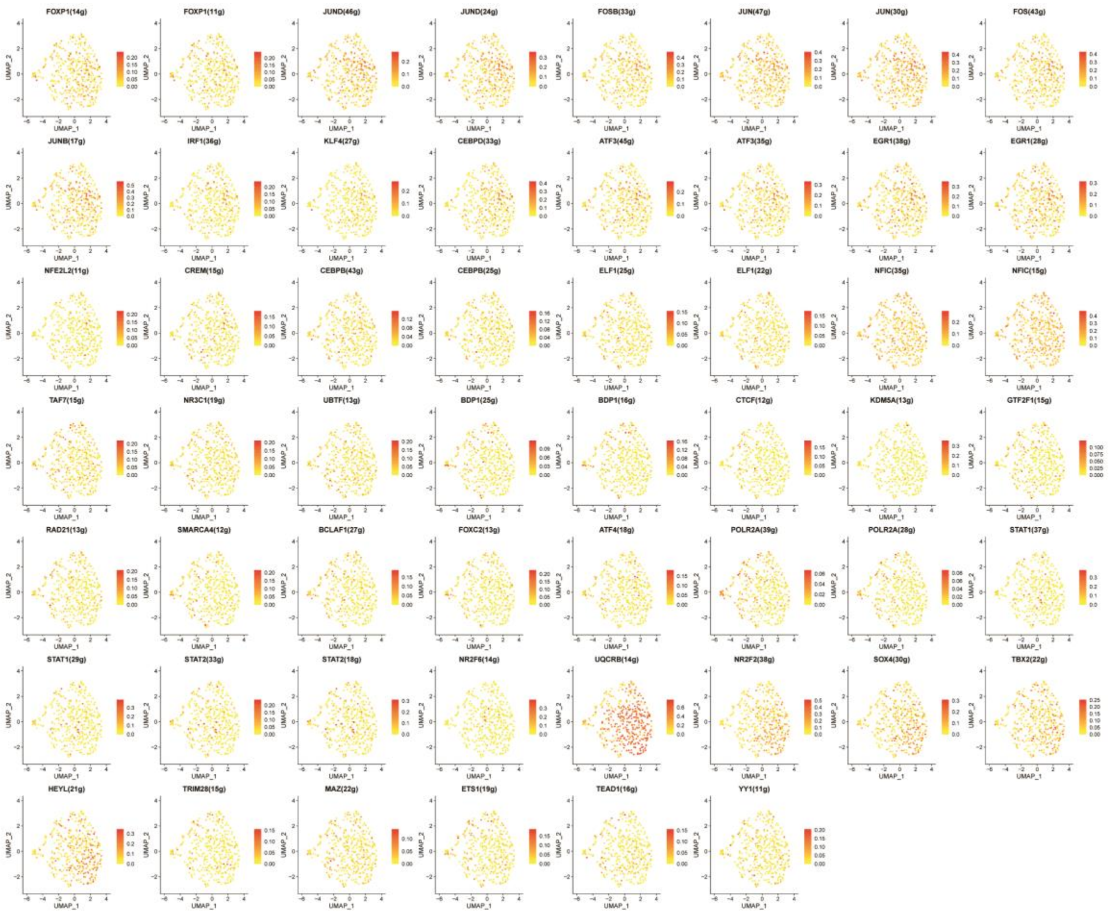

Supplementary Figure S4. Feature plots of differentially activated TFs among CAFs.

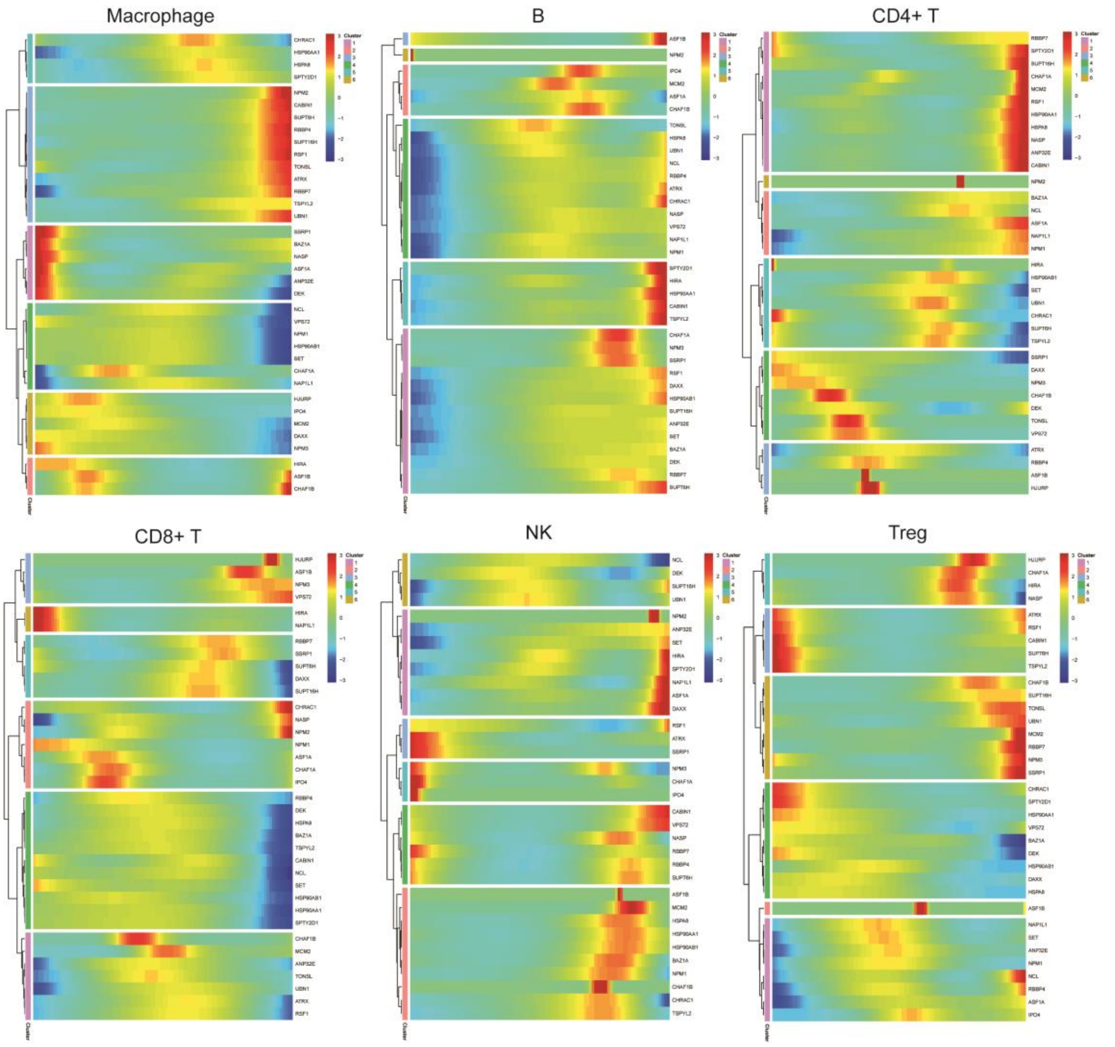

Supplementary Figure S5. Trajectory Analyses shows the role of HCs genes in macrophages, B cells, CD4+T cells, CD8+T Cells, NK cells, and Treg cells.

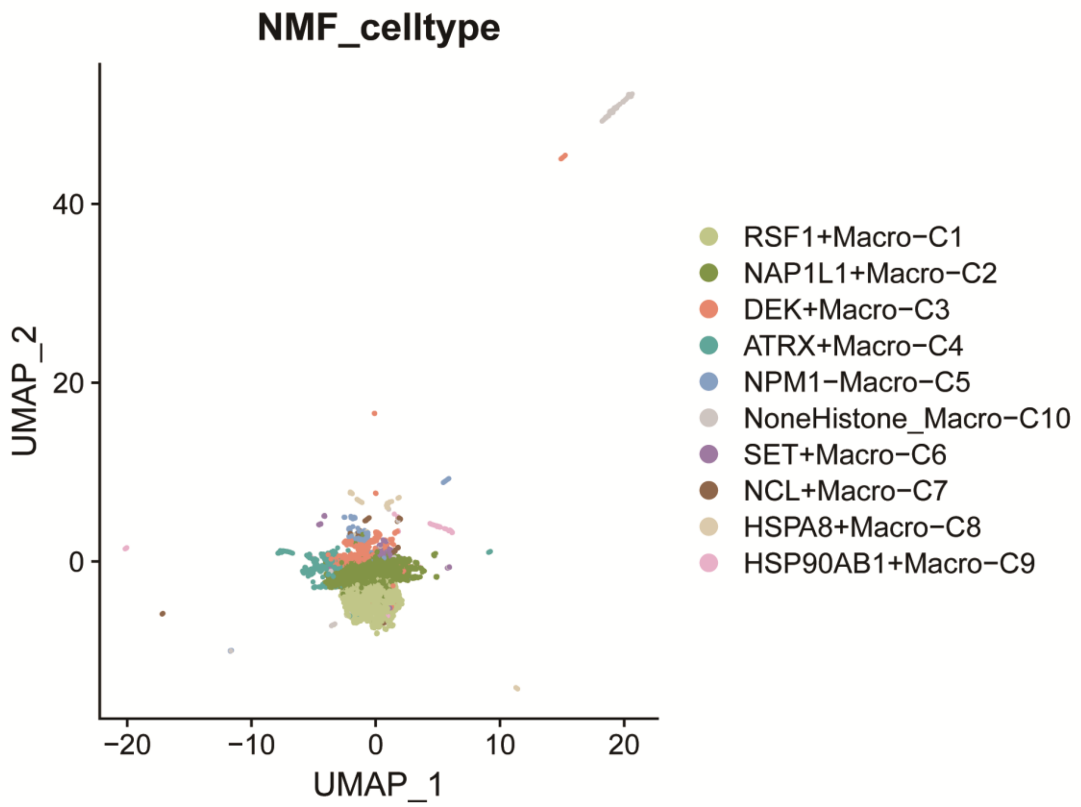

Supplementary Figure S6. UMAP plot for macrophages by 10 cell types, including RSF1+Macro-C1, NAP1L1+Macro-C2, DEK+Macro-C3, ATRX+Macro-C4, NPM1+Macro-C5, SET+Macro-C6, NCL+Macro-C7, HSPA8+Macro-C8, HSP90AB1+Macro-C9, and NoneHistone_ Macro-C10.

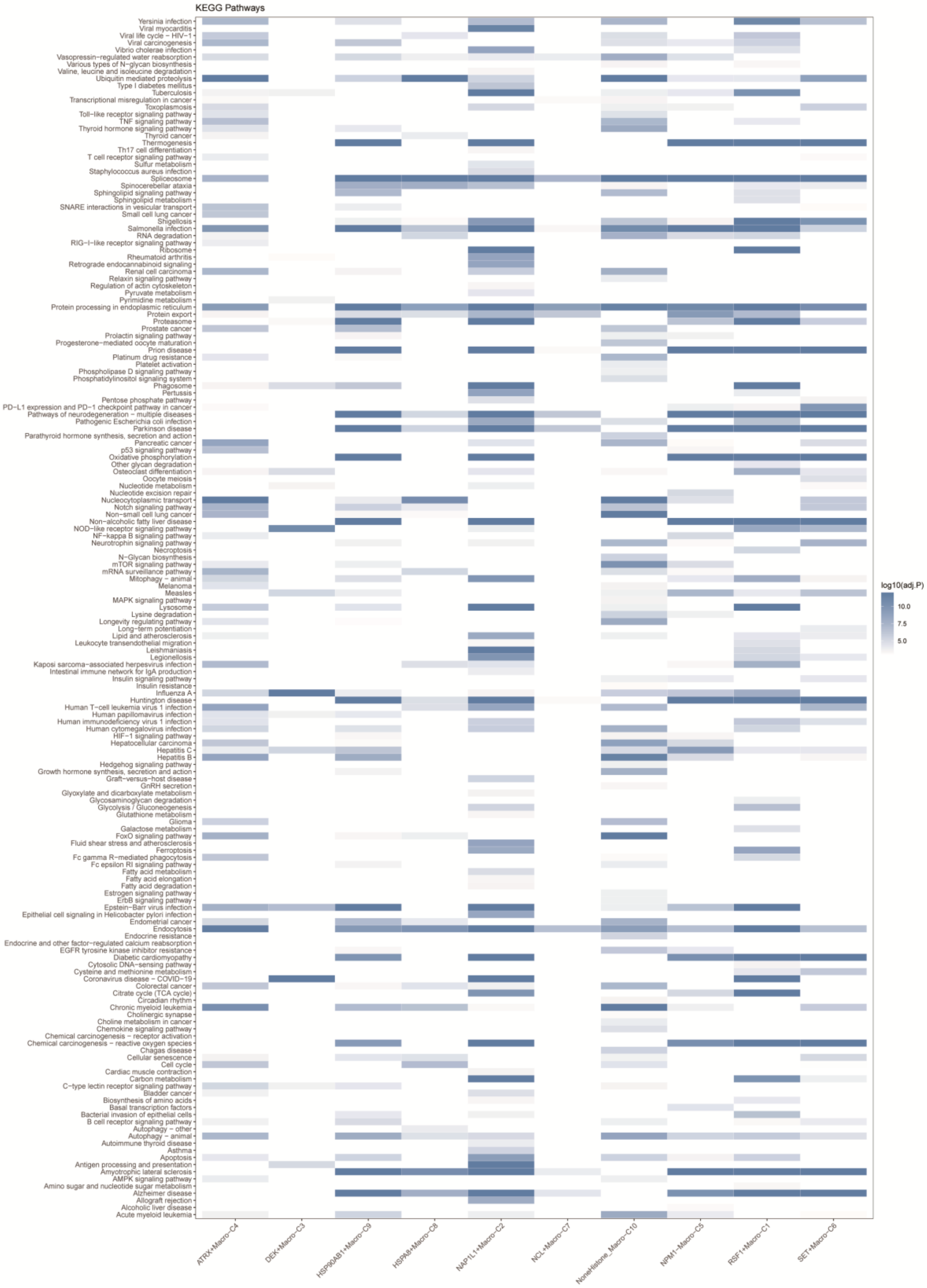

Supplementary Figure S7. Heatmap displayed the activated KEGG pathway in main HCs-Macro-clusters by utilizing the DEGs among these groups.

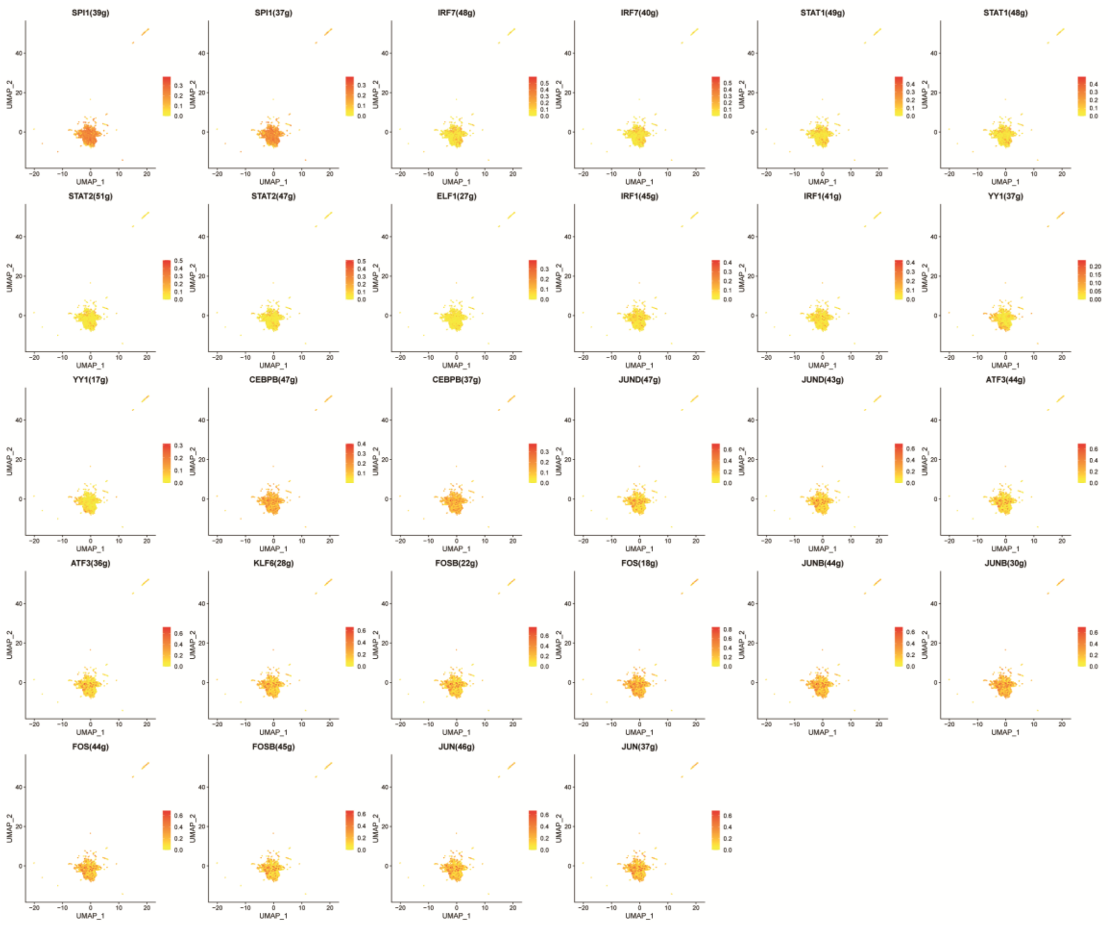

Supplementary Figure S8. Feature plots of differentially activated TFs among macrophages.

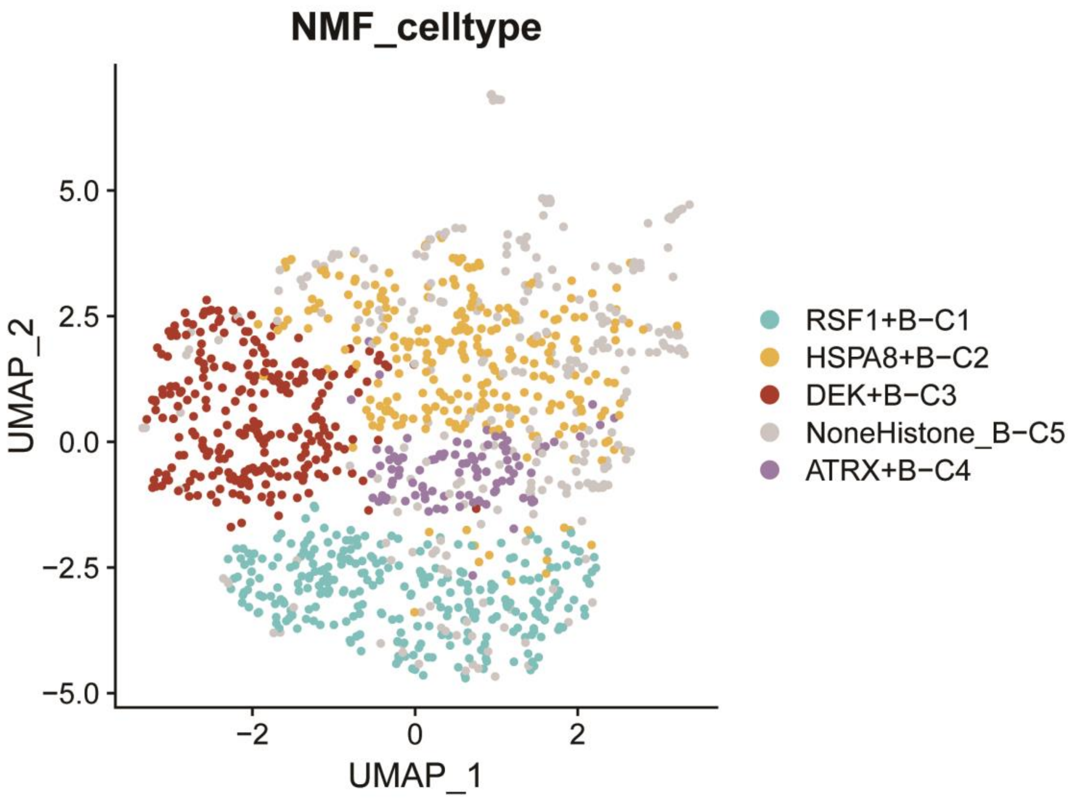

Supplementary Figure S9. UMAP plot for B cells by 5 cell types, including RSF1+B-C1, HSPA8+B-C2, DEK+B-C3, ATRX+B-C4, and NoneHistone_ B-C5.

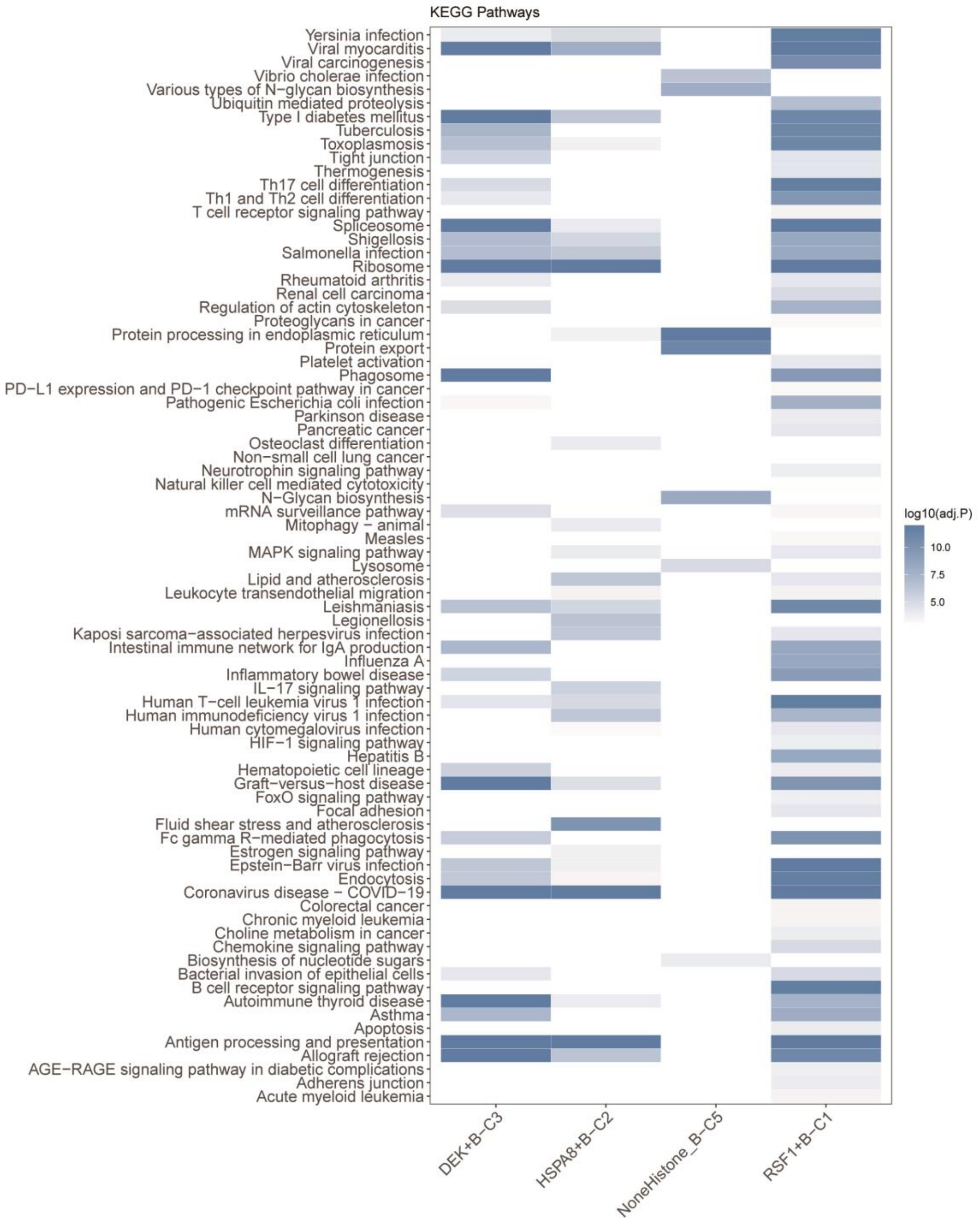

Supplementary Figure S10. Heatmap showing the activated KEGG pathway in main HCs-B-clusters by using the DEGs among these groups.

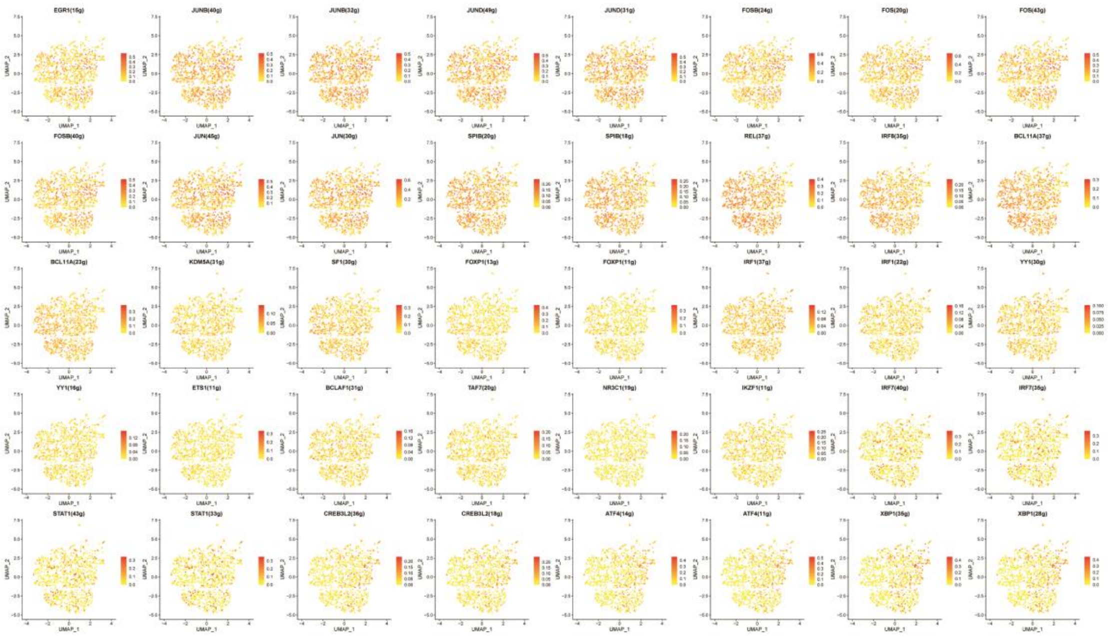

Supplementary Figure S11. Feature plots of differentially activated TFs among B cells.

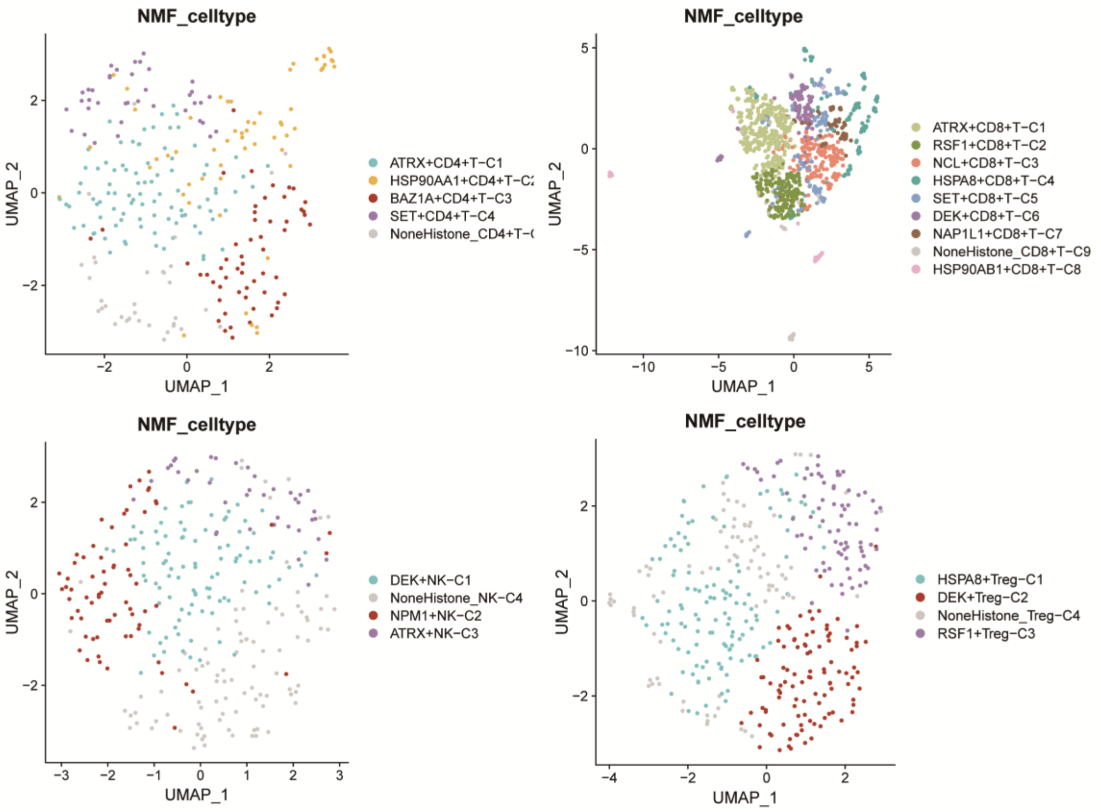

Supplementary Figure S12. UMAP plots of CD4+T cells, CD8+T cells, NK cells and Treg cells. CD4+T cells were identified into 5 cell types, including ATRX+CD4+T-C1, HSP90AA1+CD4+T-C2, BAZ1A+CD4+T-C3, SET+CD4+T-C4, and NoneHistone_CD4+T-C5. CD8+T cells were identified into 5 cell types, including ATRX+CD8+T-C1, RSF1+CD8+T-C2, NCL+CD8+T-C3, HSPA8+CD8+T-C4, SET+CD8+T-C5, DEK+CD8+T-C6, NAP1L1+CD8+T-C7, HSP90AB1+CD8+T-C8, and NoneHistone_CD8+T-C9. NK cells were identified into 5 cell types, including DEK+NK-C1, NPM1+NK-C2, ATRX+NK-C3, and NoneHistone_NK-C4. Treg cells were identified into 5 cell types, including HSPA8+Treg-C1, DEK+Treg-C2, RSF1+Treg-C3, and NoneHistone_Treg-C4.

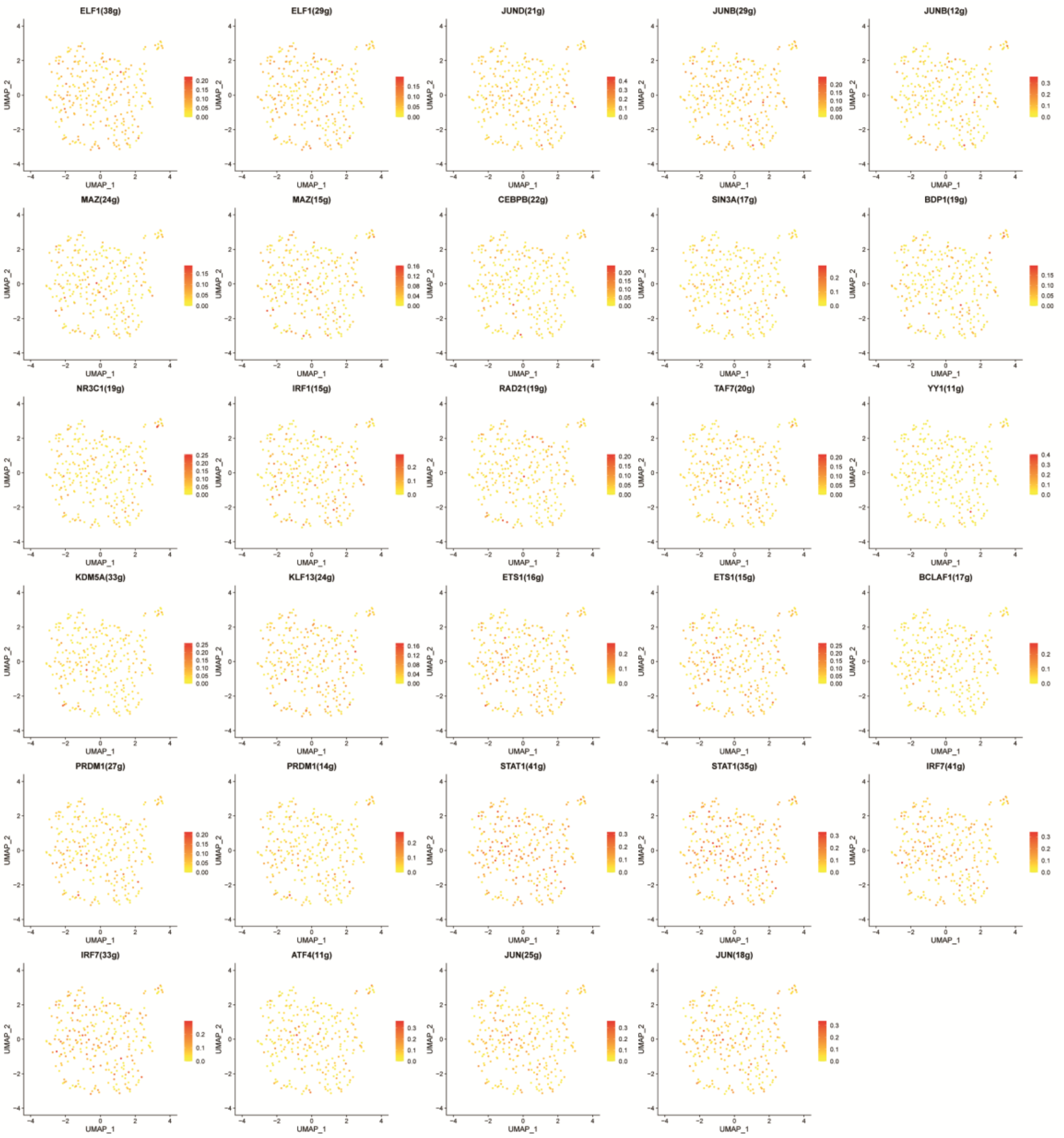

Supplementary Figure S13. Feature plots of differentially activated TFs among CD4+T cells.

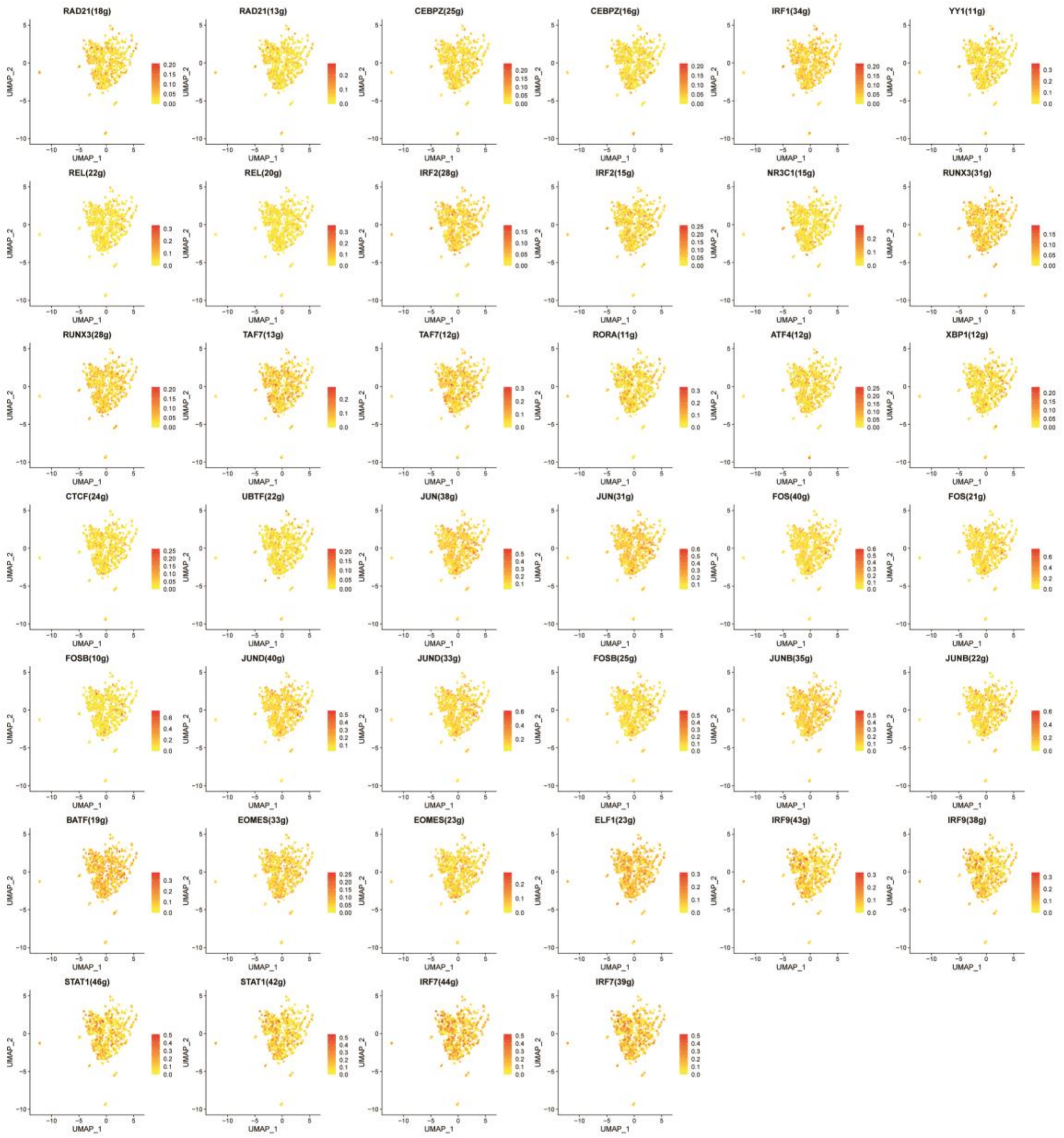

Supplementary Figure S14. Feature plots of differentially activated TFs among CD8+T cells.

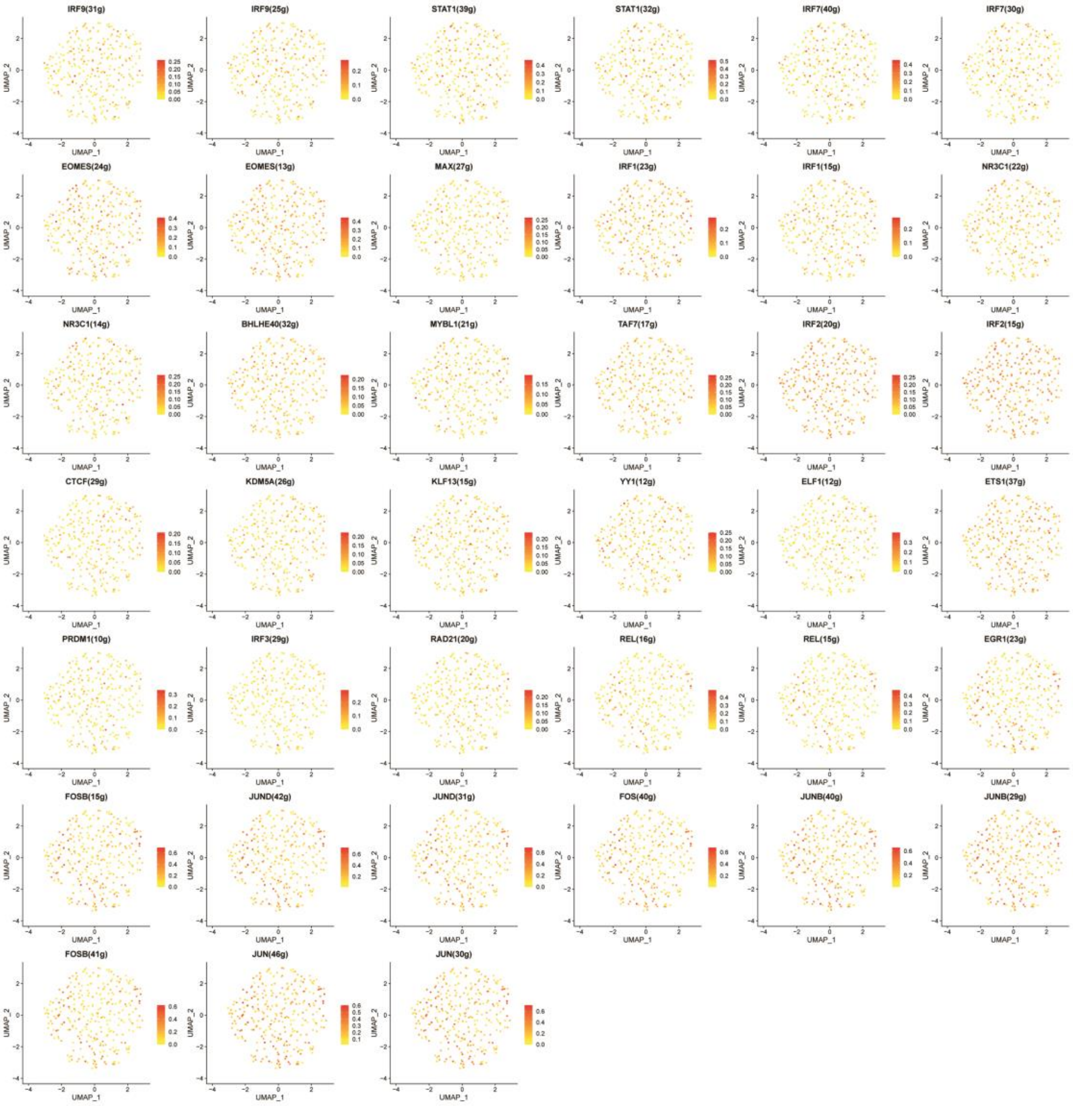

Supplementary Figure S15. Feature plots of differentially activated TFs among NK cells.

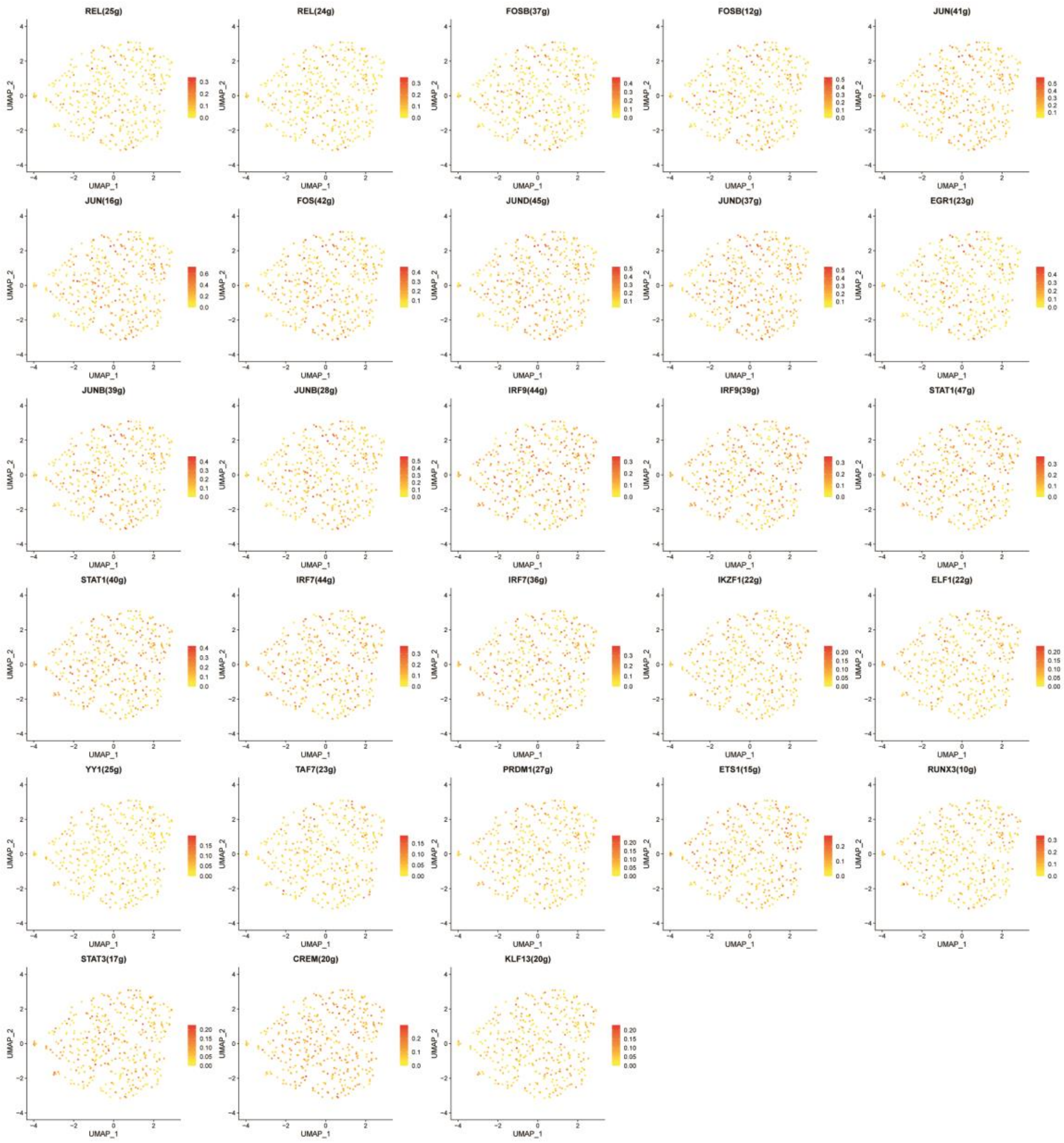

Supplementary Figure S16. Feature plots of differentially activated TFs among Treg cells.

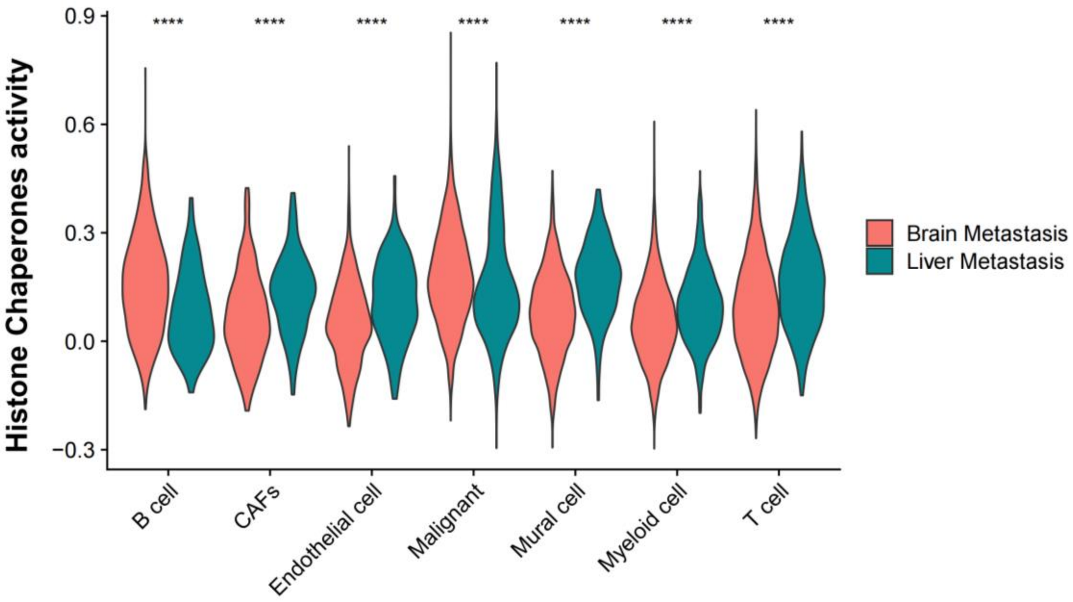

Supplementary Figure S17. Violin plots of histone chaperones activity levels among cell types between brain metastasis and liver metastasis (** p < 0.0001).**
