# Supplementary material for: Single-cell histone chaperones patterns guide intercellular communication of tumor microenvironment that contribute to breast cancer metastases
Source: Cancer Cell Int. 2023 Dec 6;23:311. doi: 10.1186/s12935-023-03166-4 (PMC10702093; doi:10.1186/s12935-023-03166-4)
Supplement: Supplementary file 2 — Supplementary Material 2 [file 12935_2023_3166_MOESM2_ESM.pdf]

Supplementary Table S1. Clinical information for metastatic breast cancer patients analyzed by scRNA-seq in this study. IDC, Invasive ductal carcinoma.

Case ID	Gender	Age	Menstrual status	Pathological type	Metastatic site	Metastasis pattern	Time to metastasis (Month)	Pre-treatment of metastatic disease
P01	Female	48	Pre-menopause	IDC	Brain	Heterochronous	53	Trastuzumab, Pertuzumab, nab-paclitaxel
P02	Female	54	Post-menopause	IDC	Brain	Heterochronous	28	/
P03	Female	41	Pre-menopause	IDC	Brain	Heterochronous	22	/
P04	Female	49	Pre-menopause	IDC	Liver	Heterochronous	56	/
P05	Female	51	Pre-menopause	IDC	Liver	Heterochronous	48	/
P06	Female	55	Pre-menopause	IDC	Liver	Heterochronous	102	/

Supplementary Table S2. The number of cells per cluster and per patient analyzed in this study.

Case ID	B cell	CAFs	Endothelial cell	Malignant	Mural cell	Myeloid cell	T cell
P01	28(0.50%)	101(1.82%)	826(14.88%)	3(0.05%)	1885(33.96%)	2316(41.73%)	391(7.05%)
P02	846(13.94%)	100(1.65%)	128(2.11%)	2132(35.12%)	520(8.57%)	998(16.44%)	1347(22.19%)
P03	184(2.62%)	39(0.56%)	49(0.70%)	2794(39.86%)	85(1.21%)	2338(33.35%)	1521(21.70%)
P04	76(0.88%)	10(0.12%)	99(1.15%)	7728(89.44%)	34(0.39%)	491(5.68%)	202(2.34%)
P05	40(0.51%)	128(1.63%)	134(1.71%)	6829(86.93%)	59(0.75%)	287(3.65%)	379(4.82%)
P06	4(0.08%)	29(0.59%)	10(0.2%)	4701(95.76%)	24(0.49%)	96(1.96%)	45(0.92%)

Supplementary Table S3. A list of 36 Histone chaperones.

ANP32E

ASF1A

ASF1B

ATRX

BAZ1A

CABIN1

CHAF1A

CHAF1B

CHRAC1

DAXX

DEK

HIRA

HJURP

HSP90AA1

HSP90AB1

HSPA8

IPO4

MCM2

NAP1L1

NASP

NCL

NPM1

NPM2

NPM3

RBBP4

RBBP7

RSF1

SET

SPTY2D1

SSRP1

SUPT16H

SUPT6H

TONSL

TSPYL2

UBN1

VPS72

Supplementary Table S4. siRNAs sequence.

Non-targeting	siControl	5'-UUCUCCGAACGUGUCACGUTT
HSPA8	siHSPA8#1	5'-GCUGGUCUCAAUGUACUUATT
	siHSPA8#2	5'-CACCAUUGAAGAGGUUGAUTT
